# Histological and transcriptomic effects of 17α-methyltestosterone on zebrafish gonad development

**DOI:** 10.1186/s12864-017-3915-z

**Published:** 2017-07-24

**Authors:** Stephanie Ling Jie Lee, Julia A. Horsfield, Michael A. Black, Kim Rutherford, Amanda Fisher, Neil J. Gemmell

**Affiliations:** 1 Department of Anatomy, University of Otago, Dunedin, Otago New Zealand; 2 Department of Pathology, University of Otago, Dunedin, Otago New Zealand; 3 Department of Biochemistry, University of Otago, Dunedin, Otago New Zealand

**Keywords:** Sex differentiation, Gonad differentiation, Androgens, Zebrafish

## Abstract

**Background:**

Sex hormones play important roles in teleost ovarian and testicular development. In zebrafish, ovarian differentiation appears to be dictated by an oocyte-derived signal via Cyp19a1a aromatase-mediated estrogen production. Androgens and aromatase inhibitors can induce female-to-male sex reversal, however, the mechanisms underlying gonadal masculinisation are poorly understood. We used histological analyses together with RNA sequencing to characterise zebrafish gonadal transcriptomes and investigate the effects of 17α-methyltestosterone on gonadal differentiation.

**Results:**

At a morphological level, 17α-methyltestosterone (MT) masculinised gonads and accelerated spermatogenesis, and these changes were paralleled in masculinisation and de-feminisation of gonadal transcriptomes. MT treatment upregulated expression of genes involved in male sex determination and differentiation (*amh*, *dmrt1*, *gsdf* and *wt1a*) and those involved in 11-oxygenated androgen production (*cyp11c1* and *hsd11b2*). It also repressed expression of ovarian development and folliculogenesis genes (*bmp15*, *gdf9*, *figla*, *zp2.1* and *zp3b*). Furthermore, MT treatment altered epigenetic modification of histones in zebrafish gonads. Contrary to expectations, higher levels of *cyp19a1a* or *foxl2* expression in control ovaries compared to MT-treated testes and control testes were not statistically significant during early gonad development (40 dpf).

**Conclusion:**

Our study suggests that both androgen production and aromatase inhibition are important for androgen-induced gonadal masculinisation and natural testicular differentiation in zebrafish.

**Electronic supplementary material:**

The online version of this article (doi:10.1186/s12864-017-3915-z) contains supplementary material, which is available to authorized users.

## Background

Natural and synthetic steroid hormones influence gonadal sex differentiation and sex ratios in teleost fish [1–5]. In particular, androgens regulate testicular development, spermatogenesis, male secondary sex characteristics, sexual behaviour and maintenance of male sexual phenotype in teleosts [4, 6–9]. The major natural androgen in teleost fish is 11-ketotestosterone [4], which is converted from androstenedione and testosterone via the steroidogenic enzymes 11β-hydroxylase (Cyp11c1) and 11β-hydroxysteroid dehydrogenase (Hsd11b2) [10–13]. The effects of these androgens are mediated by androgen receptors [10, 14].

Synthetic androgens are structural mimics of the native androgen receptor ligands testosterone and 11-ketotestosterone (11-KT). They include 17α-methyltestosterone (MT), 17α-methyldihydrotestosterone, 17β-trenbolone, mibolerone and mesterolone [15]. Treatment with exogenous androgens at the labile stage during early development triggers testicular development, stimulates precocious spermatogenesis along with phenotypic and behavioural masculinisation, skews sex ratios towards males in gonochoristic fish [16–24] and accelerates female-to-male sex change in protogynous hermaphroditic fish [25–29]. Studies suggest that androgen-induced female-to-male phenotypic sex reversal is functional and persistent [30–33]. Androgen induction of female-to-male sex reversal [15, 34] is routinely used for sex control in aquaculture [35], with MT being the most frequently used androgen for production of all-male populations in commercial settings [15] because of its high masculinising potency [15].

Two hypotheses exist for the mode of action of androgens for induction of testicular differentiation: (1) indirect, via aromatase inhibition which reduces estrogen production [36–38] and (2) direct, via interactions with androgen receptor [36]. Prior studies of androgen-induced gonadal masculinisation in European seabass [39], Japanese flounder [19], groupers [26, 29], Nile tilapia [40] and rainbow trout [41–45] using candidate gene and microarray approaches [46] support the aromatase inhibition hypothesis. The gonadal masculinisation observed in zebrafish following treatment with aromatase inhibitor also strongly supports this hypothesis [38]. However, recent studies in tilapia and grouper implicate androgen receptor in androgen-induced phenotypic masculinisation [36, 47]. Overall, the molecular mechanisms underpinning androgen-induced gonadal masculinisation in fishes remain to be clarified.

Zebrafish (*Danio rerio*) is a popular toxicological model and has been used extensively to study the effects of endocrine-disrupting chemicals on gonad development and sexual phenotype [24, 30–32, 48–57]. The primary sex determining region present in wild zebrafish strains appear to have largely been lost in laboratory strains through the domestication process [58]. Almost all zebrafish in the world are derived from domesticated strains, with most from the AB strain and its derivatives except for the WIK strain. Domesticated zebrafish use a polygenic sex determination system [59] vulnerable to the effects of high temperature [60, 61], hypoxia [62], rearing density [63, 64], inbreeding, out-crossing [65, 66] and hormones [30]. Zebrafish is a juvenile hermaphrodite, wherein all individuals initially develop a non-functional ‘juvenile ovary’ [67–70]. Oocytes undergo apoptosis in juvenile ovaries of presumptive males during juvenile ovary-to-testis gonadal transformation [71]. In contrast, oocytes continue oogenesis and oocyte maturation in presumptive females [68] via unknown ovarian aromatase (Cyp19a1a)-mediated mechanisms [70]. Depending on the dosage, treatment with exogenous androgens can induce female-to-male sex reversal [24, 30–33] and paradoxical feminisation in zebrafish [72]. The *Tg(vas:egfp)* zebrafish line exhibits sexually dimorphic expression of green fluorescent protein which facilitates in vivo sex identification during early gonad development [73]. It has been used to study estrogenic effects in zebrafish [74].

Little is known about the direct transcriptional effects of MT during gonad masculinisation. In this study, we treated juvenile *Tg(vas:egfp)* transgenic zebrafish with 100 ng/L MT from 20 days post fertilisation (dpf) to 40 dpf and 60 dpf. These time points were selected to reflect the onset (20 dpf) and completion (40 dpf) of juvenile ovary-to-testis transformation as well as the onset of female puberty (60 dpf) in zebrafish [68, 69, 75]. RNA sequencing (RNA-Seq) was used to profile global gene expression patterns in MT-treated gonads. The gonadal transcriptomes of MT-treated zebrafish were compared with those of control zebrafish testes and ovaries to provide insights into the molecular basis for MT-induced gonadal masculinisation in zebrafish.

## Methods

### Ethics statement

This study was approved by the University of Otago Animal Ethics Committee (AEC No. 101/09). All experiments were performed in accordance with the Good Practice Guide for the use of animals in research, testing and teaching.

### Zebrafish husbandry

Zebrafish were maintained according to Westerfield [76]. We used larval and juvenile transgenic zebrafish expressing an enhanced green fluorescent protein (EGFP) under the control of the *vasa* promoter, *Tg(vas:egfp)*, derived from the domesticated Tübingen/AB strains [73]. EGFP expression in *Tg(vas:egfp)* transgenic zebrafish enables visualisation and isolation of gonads before the gonads can be unequivocally distinguished from other tissues [77]. Higher levels of fluorescence are detected in ovaries than testes [69, 73], which can be used to distinguish the phenotypic sex of the fish.

### 17α-methyltestosterone treatment

17α-methyltestosterone (MT) was purchased from Sigma Aldrich (Sigma-Aldrich Sweden AB, Stockholm, Sweden) and dissolved in 100% ethanol to prepare stock solutions of 50 mg/L. At 18 to 19 dpf, juvenile zebrafish were transferred from 4 L tanks into petri dishes where they were size-sorted by visual inspection. Zebrafish that were unusually small (<5.5 mm) or large (>8.5 mm) were removed. The remaining juvenile zebrafish (between 5.5 to 8.5 mm in length) were transferred into 4 L tanks containing 3.5 L of system water at a density of 20 individuals per tank.

For the MT exposure, the juvenile zebrafish were exposed to system water containing either 100 ng/L of MT dissolved in 0.0001% ethanol (100 ng/L MT) or 0.0001% ethanol alone (solvent control). Each exposure group consisted of 3 biological replicates comprising 20 juvenile zebrafish each. The exposure was performed continuously under a semi-static system for 20 or 40 days extending from 20 dpf to 40 dpf or 60 dpf. Water with equivalent concentrations of MT and ethanol was used to replace half of the water in each beaker every second day for the MT treatment and solvent control groups respectively.

### Determination of gonadal morphology and sex ratios

At the termination of the treatment at 40 dpf and 60 dpf, the juvenile zebrafish were sacrificed via snap chilling in ice water. EGFP expression of each zebrafish was observed under a Leica M205 FA fluorescence dissecting stereo microscope (Leica Microsystems, Bannockburn, Illinois, USA) to determine the gonadal sex of each fish and sex ratios for each exposure group. Between 18 to 24 fish per exposure group (comprising 6 to 8 fish from each of the 3 replicates per time point) were selected for histological analysis to confirm gonadal sex as determined by EGFP expression and to determine the developmental stages of each gonad. Whole zebrafish were fixed overnight in 10% neutral buffered formalin, dehydrated and embedded in paraffin. Haematoxylin and eosin-stained sections were examined under light microscopy to determine gonadal sex. Ovaries were classified as Stage I or Stage II [75], based on the absence or presence of Stage II oocytes containing cortical alveoli, respectively [78]. Testes were categorised into transition gonads, immature testes or mature testes. Gonads with basophilic apoptotic bodies and infiltration of stromal tissue were classified as transition gonads [68]. Testes were classified as immature or mature depending on the absence or presence of spermatozoa [79]. Spermatids differentiate into spermatozoa, through a series of morphological changes inclusive of nuclear compaction and flagellum formation, which are released into the seminiferous tubule lumen at the end of spermiogenesis [80].

### RNA isolation

The juvenile zebrafish gonads were dissected and placed into aliquots of RNAlater (Invitrogen, ﻿﻿Life Technologies GmbH / ThermoFisher Scientific﻿﻿, Darmstadt, Germany) or Lysis buffer (RLT buffer from an RNeasy Mini kit, Qiagen GmbH, Hilden, Germany or RA1 lysis buffer from a Nucleospin RNA II kit, Macherey-Nagel ﻿﻿﻿﻿GmbH & Co. KG﻿﻿, Düren﻿﻿, Germany) and stored at −80 °C for subsequent RNA isolation. The gonads from ten individuals per exposure group were pooled together for RNA extraction. Gonads were homogenized by passing through a 20 gauge needle or using 2 cycles of the TissueLyser II system (Qiagen GmbH, Hilden, Germany) set at 20 Hz for 1 min per cycle.

RNA was isolated using an RNeasy Mini kit (Qiagen GmbH, Hilden, Germany) or a Nucleospin RNA II kit (Macherey-Nagel ﻿﻿GmbH & Co. KG﻿﻿, Düren, Germany) and eluted in 53ul of RNase-free water.

**Fig. 1:**
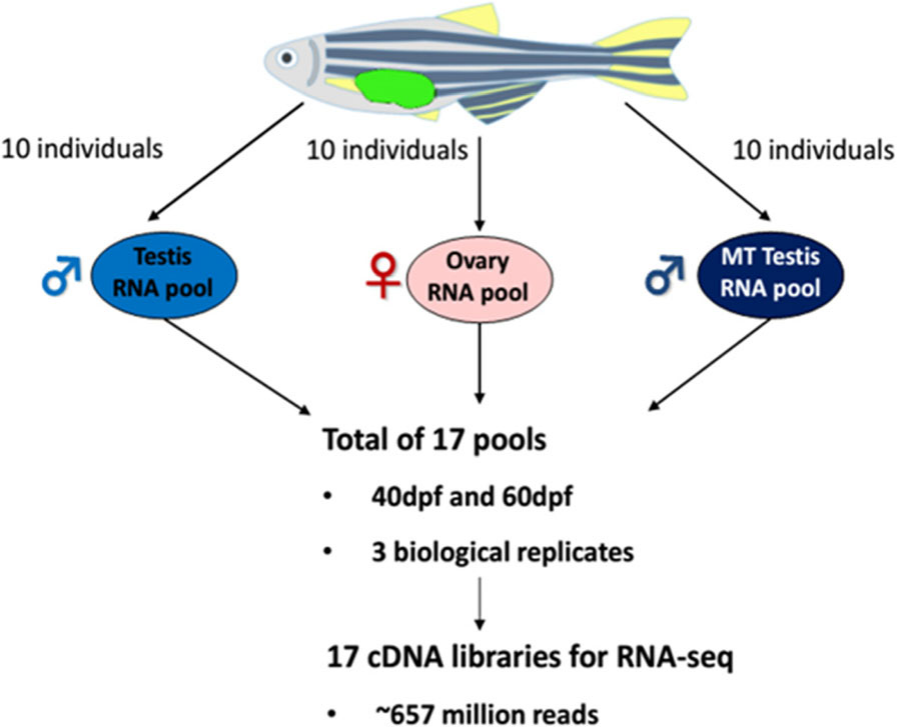
General overview schematic of the zebrafish gonad RNA-Seq experiment. RNA was isolated from pools of gonads dissected from 10 individuals and subjected to RNA-Seq analyses. The gonads were pooled according to sex (male, female and masculinised male), developmental period tested (40 dpf and 60 dpf) and treatment group (solvent control and 17α-methyltestosterone treatment). Gonad RNA pools were analysed in triplicate

Three biological replicates of gonad pools consisting of 10 individuals each were generated for each exposure group (MT exposure and solvent control), sex (male or female) and time point (40 dpf or 60 dpf) (Fig. 1). The RNA samples were quantified and integrity was assessed using the Agilent 2100 Bioanalyser (Agilent Technologies, Palo Alto, California, USA). RNA samples with RNA Integrity Numbers (RIN) ≥7.0 (majority with RIN ≥ 8.0 to 9.0) were used for RNA sequencing (RNA-Seq).

### RNA sequencing

The Illumina TruSeq RNA sample preparation kit v2 (Illumina, Inc., San Diego, California, USA) was used with 1 μg of starting total RNA from the gonad pools to generate 100 bp paired end (PE) cDNA libraries in accordance to the manufacturer’s instructions. Three biological replicates were generated for each exposure group except for the 40 dpf MT-treated testis group, which was limited to two biological replicates. A total of seventeen gonad tagged cDNA libraries were sequenced: 40CO1–3 (40 dpf control ovary pools 1 to 3), 40CT1–3 (40 dpf control testis pools 1 to 3), 40MT1–2 (40 dpf MT-treated testis pools 1 and 2), 60CO1–3 (60 dpf control ovary pools 1 to 3), 60CT1–3 (60 dpf control testis pools 1 to 3), and 60MT1–3 (60 dpf MT-treated testis pools 1 to 3), on a HiSeq 2000 sequencer (Illumina, Inc., San Diego, California, USA) at the New Zealand Genomics Limited Otago facility (Otago Genomics and Bioinformatics Facility, University of Otago, Dunedin,﻿ Otago, New Zealand) (Table 1). The raw sequencing reads have been submitted to NCBI Sequence Read Archive (SRA; http://www.ncbi.nlm.nih.gov/sra) repository under accession number SRP102493.

**Table 1:** Gonad cDNA libraries generated for RNA sequencing

Stage	Treatment	Gonad	Replicates	Replicate names
40 dpf	Solvent control	Ovary	3	40CO1, 40CO2, 40CO3
40 dpf	Solvent control	Testes	3	40CT1, 40CT2, 40CT3
40 dpf	17α- methyltestosterone	Testes	2	40MT1, 40MT2
60 dpf	Solvent control	Ovary	3	60CO1, 60CO2, 60CO3
60 dpf	Solvent control	Testes	3	60CT1, 60CT2, 60CT3
60 dpf	17α- methyltestosterone	Testes	3	60MT1, 60MT2, 60MT3

### Read annotation, mapping, assembly and quantification

The quality of the raw reads was assessed using the FastQC software [81] and the sequencing QC report tool in CLC Genomics Workbench 6.0.2 software (Qiagen Bioinformatics GmbH, Hilden, Germany). CLC Genomics Workbench was used to trim low quality sequences (phred-based error probability threshold of 0.05) and to remove TruSeq adapter sequences from the raw reads. Trimmed reads from each pool were mapped and aligned to the Zebrafish Zv9 reference sequence (ftp://ftp.ensembl.org/pub/current_fasta/danio_rerio/dna/) from the ENSEMBL database. Annotation of the Zebrafish ZV9 reference genome was performed using the respective GTF file (ftp://ftp.ensembl.org/pub/current_gtf/danio_rerio/) using the CLC Genomics Workbench 6.0.2 ‘Annotate with GFF/GTF’ plug-in. In order to correct for differences in transcript length and library size, transcript abundance was normalised using the reads per kilobase per million mapped reads (RPKM) method [82]. Transcripts with RPKM value greater than or equal to 1.0 (RPKM ≥1.0) were regarded as expressed. Transcripts with RPKM value greater than or equal to 5.0 (RPKM ≥5.0) were regarded as reliably expressed.

### Identification of differentially expressed transcripts

Differentially expressed transcripts were identified in the zebrafish gonad transcriptomes across different conditions (sex, age, MT treatment and gonad phenotype) using the proportions-based beta-binomial Baggerley’s test [83]. The RPKM data was transformed by adding a constant (1.0) and then normalised using a quantile normalisation. Transcripts responsive to MT were identified via pairwise comparisons of MT-treated gonads with age-matched control ovaries and testes. Transcripts were regarded as differentially expressed if they complied with (1) the normalised fold change threshold of greater than or equal to two for upregulated transcripts and smaller than or equal to minus two for downregulated transcripts (≥ 2.0-fold) and (2) a false discovery rate (FDR) corrected *p*-value of ≤0.05 (*p* ≤ 0.05) [84].

To investigate the overall gene expression patterns across the treatment conditions and phenotypic sexes, we performed hierarchical clustering using Pearson correlation distance with complete linkage, and Principal Component Analyses (PCA), as implemented and visualised in CLC Genomics Workbench. The sets of differentially expressed transcripts that overlapped or differed between treatments were visualised using Venn diagrams plotted using the Venny software (http://bioinfogp.cnb.csic.es/tools/venny/).

### Gene ontology enrichment analysis of differentially expressed genes

Gene Ontology Consortium (GO) [85] functional annotation terms and categories were assigned to the differentially expressed genes with the ‘Add Annotations’ tool of CLC Genomics Workbench. The GO terms, IDs and annotations were downloaded from the Gene Ontology database in the 20 May 2013 release of the ZFIN Zebrafish GO gene association file (http://www.geneontology.org/GO.downloads.annotations.shtml).

### Functional enrichment and network pathway analysis

Biological functions and metabolic pathways significantly overrepresented among the differentially expressed genes were identified using Metacore (GeneGo, Thomson Reuters, Carlsbad, California, USA). This approach utilizes Fisher’s exact test with an FDR correction for multiple testing. Differentially expressed genes were defined as genes where (1) the absolute value for normalised fold change was greater than or equal to two and (2) the FDR adjusted *p*-value was less than 0.05 [84].

### Validation of differentially expressed genes using quantitative RT-PCR

Quantitative real-time PCR (qRT-PCR) was conducted on 12 differentially expressed transcripts identified from the RNA-seq data. TaqMan Gene Expression Assays (Applied Biosystems, Thermo Fisher Scientific, Waltham, Massachusetts, USA) specific to our genes of interest were used with a Stratagene Mx3000P (Agilent Technologies, Santa Clara, California, USA) Real Time-PCR thermal cycler. Each qPCR reaction was performed in triplicate. *β-actin 1*, ribosomal protein L13 alpha (*rpl13α*) and eukaryotic translation elongation factor 1 alpha 1, like 1 (*eef1a1l1*) were tested for their utility as the reference genes for this study. Previous studies have shown that the expression levels of these genes remain fairly constant across different developmental periods, treatment conditions, sexes and tissue types in zebrafish [86, 87]. In this study, minor differences in *β-actin 1* expression were found between different pooled ovary samples however the expression levels of *rpl13α* and *eef1a1l1* were consistent across different tissue types (adult testes and ovaries) as previously reported [88] (data not shown). *eef1a1l1* gave the highest average expression stability values with the geNorm algorithm [89].The comparative CT method (ΔΔCt) was used to determine relative gene expression compared to the reference gene *eef1a1l1*. The female ovary groups (40CO and 60CO) were used as the calibrator for calculation of relative expression. Relative expression was expressed as fold change (Fold Change = 2 − ΔΔCt). Complete details of the genes selected, TaqMan Gene Expression Assays and qPCR cycling conditions are provided in Additional file 1.

## Results

### Sex ratios and gonad histology of control zebrafish

Sex ratios were determined via visualisation of EGFP fluorescence intensity of *Tg(vas:egfp)* zebrafish under a fluorescence microscope. Gonads exhibiting low and high EGFP fluorescence were classified as testes and ovaries respectively [69, 73]. Male and female zebrafish were present in the control at 40 dpf and 60 dpf. The control sex ratios were 32% males at 40 dpf and 45% males at 60 dpf (Additional file 2). The mortality rates were very low (≤10%) for the control with no significant difference (*p*-value > 0.05) observed between replicates (Additional file 2).

To determine the effects of MT on zebrafish gonad development, we treated juvenile zebrafish with 100 ng/L MT from 20 dpf to 40 dpf and 60 dpf. All MT-treated zebrafish had testes except for one individual at 40 dpf (40 dpf: 59/60 individuals and 60 dpf: 98/98 individuals) (Additional file 2) suggesting that MT treatment had stimulated gonadal masculinisation.

**Fig. 2:**
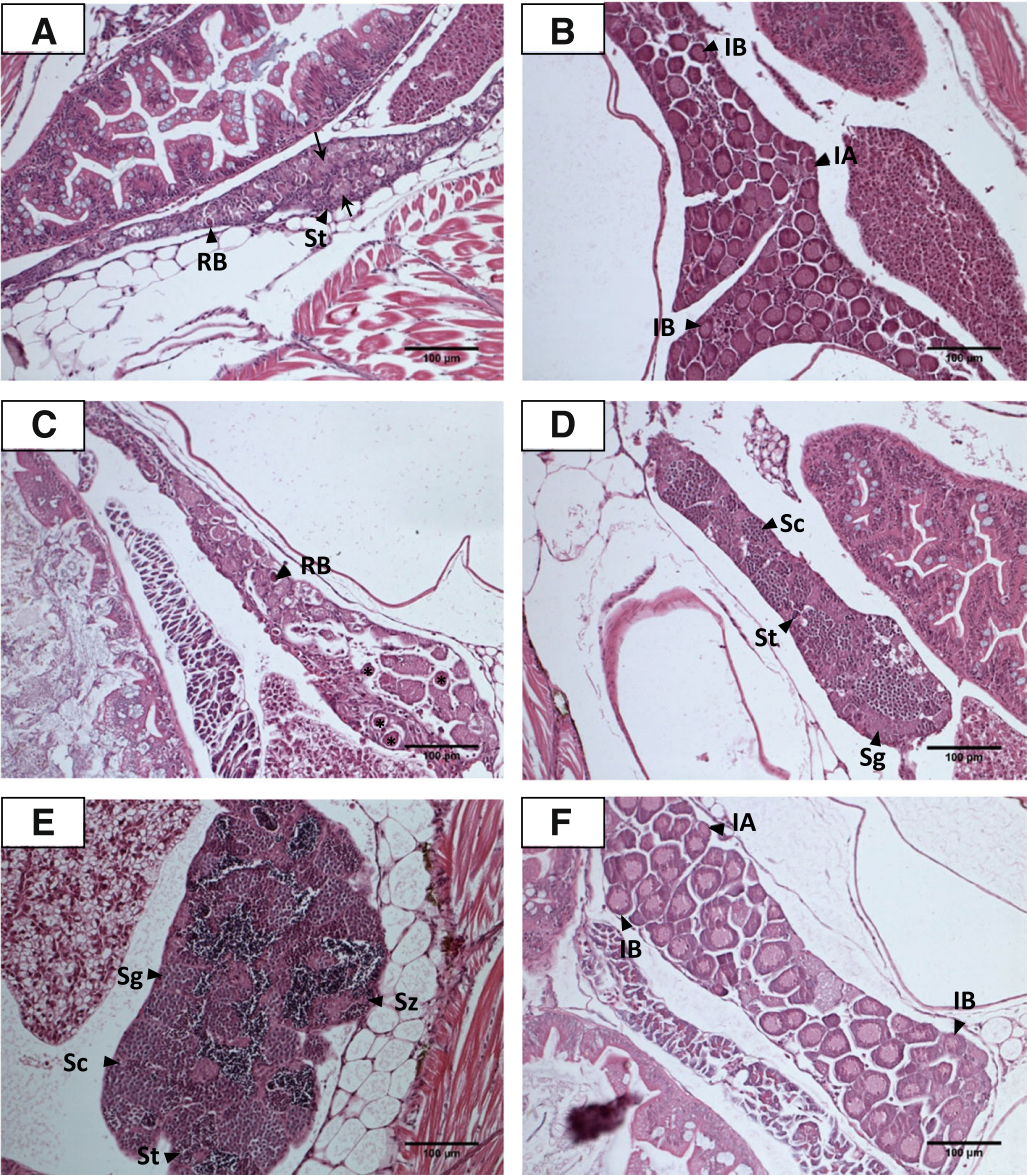
Histological analysis of gonads of 40 dpf control (**a** and **b**) and MT-treated juvenile zebrafish (**c-f**). **a** Presumptive testis characterised by the presence of residual bodies (RB), an indicator of oocyte degeneration, infiltration of stromal (Str) cells and spermatogenic tubule-like structures (→). **b** Ovary with Stage IA (IA) and Stage IB (IB) oocytes. **c** Transforming gonad. **d** Immature testis with spermatogonia (Sg), spermatocytes (Sc) and spermatids (St). **e** Mature testis possessing spermatogonia (Sg), spermatocytes (Sc), spermatids (St) and spermatozoa (Sz). **f** Ovary with Stage IA (IA) and Stage IB (IB) oocytes. Bar = 100 μm

The EGFP fluorescence signal was considerably fainter in 40 dpf testes than for 40 dpf ovaries. A few gonads (4/4 males) appeared to be undergoing gonadal transformation into testes at 40 dpf and were classified as presumptive testes (Fig. 2a). Several residual bodies indicative of apoptotic oocytes were present. The remaining oocytes in these gonads had lost their tight contact and showed signs of degeneration including shrinkage, irregularity of shape and basophilic cytoplasm. Stromal tissue had infiltrated the inter-oocyte spaces. In a few individuals, the germ cells had re-arranged to form tubule-like structures.

Regardless of the stage of ovarian development, intense EGFP fluorescence signals were detected in ovaries of 40 dpf and 60 dpf zebrafish. At 40 dpf, all control females analysed using histological examination possessed ovaries at early stages of development (14 /14 individuals). These immature ovaries were predominantly filled with tightly packed Stage IB oocytes although pre-meiotic Stage IA oogonia were located at the caudal edges of the ovaries (Fig. 2b). Most MT-treated zebrafish had mature testes at 40 dpf (17/22 individuals) (Fig. 2e). One was undergoing juvenile ovary-to-testis transformation (Fig. 2c). The remaining three individuals possessed immature testes (Fig. 2d). In contrast, control males had testes which had just completed juvenile ovary-to-testis transformation (4/4 individuals) (Fig. 2a).

Similar to 40 dpf control ovaries, the 40 dpf MT-treated ovary contained Stage IB oocytes (Fig. 2f). Early range-finding experiments testing different periods of MT exposure suggested that occasionally a few individuals exposed to MT from 20 dpf to 40 dpf or 50 dpf were refractory to the masculinising effects of MT treatment (data not shown).

**Fig. 3:**
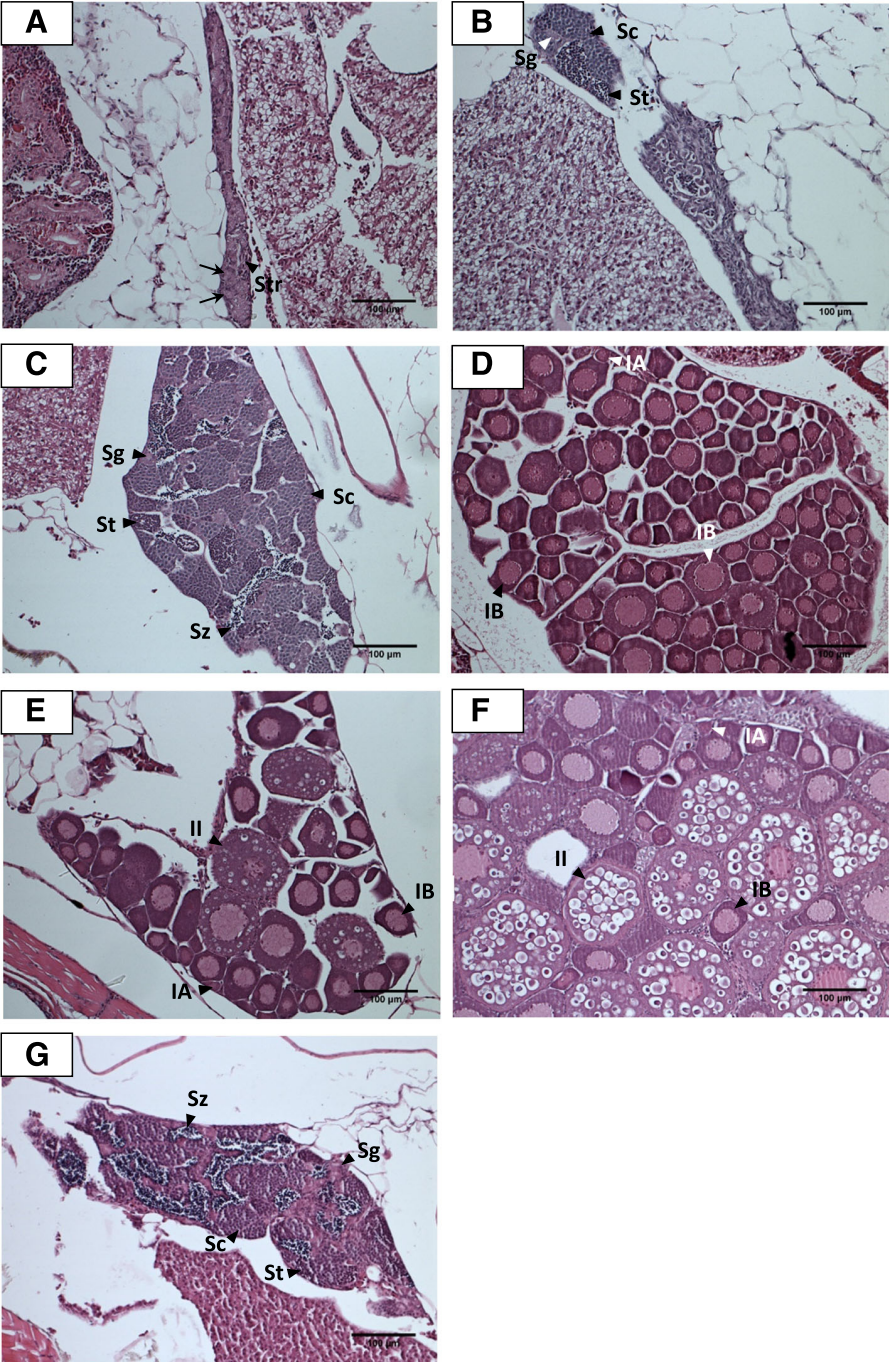
Histological analysis of gonads of 60 dpf untreated control (**a** - **f**) and MT-treated juvenile zebrafish (**g**). **a** Presumptive testis comprising infiltration of stromal (Str) cells and spermatogenic tubule-like structures (→). **b** Immature testis containing spermatogonia (Sg), spermatocytes (Sc) and spermatids (St). **c** Mature testis containing spermatogonia (Sg), spermatocytes (Sc), spermatids (St) and spermatozoa (Sz). **d** Ovary with Stage IA (IA) and Stage IB (IB) oocytes. **e** Ovary with Stage I IA (IA), Stage IB (IB) and Stage II (II) oocytes. **f** Ovary with Stage I IA (IA), Stage IB (IB) and Stage II (II) oocytes. **g** Mature testis possessing spermatogonia (Sg), spermatocytes (Sc), spermatids (St) and spermatozoa (Sz). Bar = 100 μm

Testes at 60 dpf barely exhibited any EGFP fluorescence. There were three types of 60 dpf control testes: presumptive (2/10 males), immature (4/10 males) and mature testes (4/10 males) (Fig. 3a – c). 60 dpf presumptive testes resembled 40 dpf presumptive testes which were populated with a few degenerative oocytes and large volumes of stromal tissue (Fig. 3a). Most 60 dpf control testes (8/10 individuals) were at a more advanced stage of differentiation than those at 40 dpf (Fig. 3b and c). Well-differentiated spermatogonial cysts containing spermatogonia, spermatocytes and spermatids were observed in 60 dpf control immature and mature testes (Fig. 3b and c). The lumens of mature 60 dpf control testes were filled with mature spermatozoa (Fig. 3c).

Considerable individual differences in the extent of ovarian development were seen in 60 dpf control females. A few ovaries (6/13 females) consisted primarily of Stage IB oocytes (Fig. 3d). Others (7/13 females) contained oocytes at more advanced stages of development such as Stage II cortical alveolar oocytes (Fig. 3e and f).

Greater numbers of 60 dpf MT-treated zebrafish possessed mature testes (21/21 individuals) (Fig. 3g) than control males (17/22 individuals) whose testes were at various stages of spermatogenesis (Fig. 3a - c).

### Global sex differences in gonadal gene expression patterns

To elucidate the genes involved in zebrafish sexual differentiation, we compared the transcriptomes of developing zebrafish testes and ovaries using RNA Sequencing. Gonads were classified as testes or ovaries on the basis of EGFP expression in the juvenile *Tg(vas:egfp)* zebrafish as described above. Greater numbers of gene transcripts were present in the zebrafish testis than zebrafish ovary at both 40 dpf and 60 dpf (Additional file 3). About 12,000 transcripts were consistently expressed (RPKM ≥ 5) in zebrafish testes (Table 2). In contrast, ~8000 transcripts were expressed in zebrafish ovaries at 40 dpf and 60 dpf (Table 2).

**Table 2:** Average expression levels (RPKM values) in developing gonads at 40 dpf and 60 dpf. RPKM ≥1 was defined as the RPKM threshold for expressed transcripts. RPKM ≥5 was defined as the RPKM threshold for reliable detection of expressed transcripts. RPKM, reads per kilobase per million mapped reads. 40CO, 40 dpf control ovaries. 40CT, 40 dpf control testes. 40MT, 40 dpf MT-treated testes. 60CO, 60 dpf control ovaries. 60CT, 60 dpf control testes. 60MT, 60 dpf MT-treated testes

Group	RPKM ≥ 1	RPKM ≥ 5
40CO	12,617	8047
60CO	12,269	8191
40CT	18,906	11,692
60CT	19,016	11,975
40MT	18,311	11,489
60MT	19,028	11,865

**Fig. 4:**
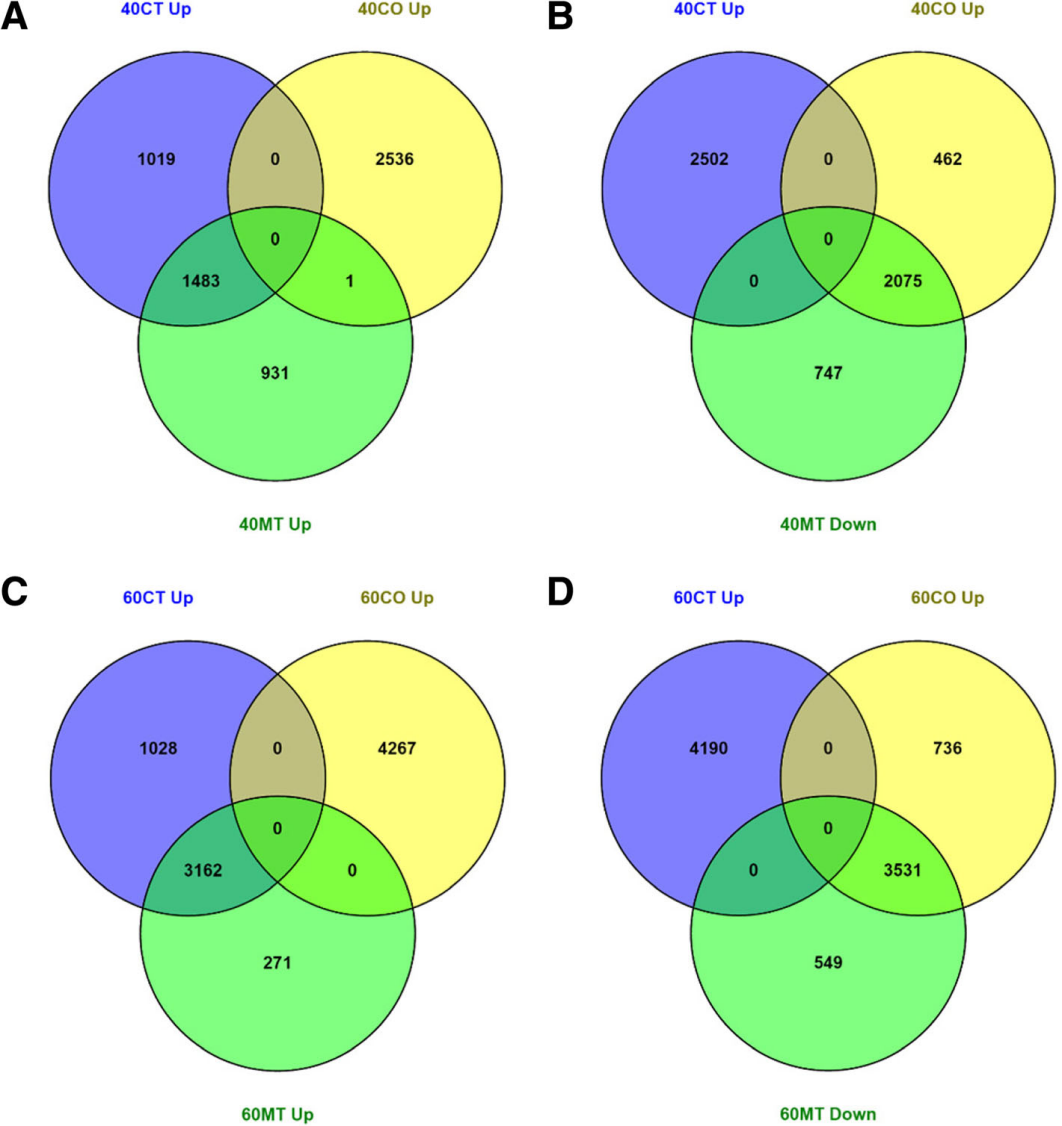
Comparison of differentially expressed genes in MT-treated zebrafish testes with testis-enriched and ovary-enriched genes of control zebrafish ovaries and testes. *n* = 3 pools of 10 individuals each. **a** Venn diagram of numbers of genes which showed upregulated expression in 40 dpf MT-treated testes (40MT) overlapped with 40 dpf testes (40CT) and 40 dpf ovaries (40CO). **b** Venn diagram of numbers of genes which showed downregulated expression in 40 dpf MT-treated testes (40MT) overlapped with 40 dpf testes (40CT) and 40 dpf ovaries (40CO). **c** Venn diagram of numbers of genes which showed upregulated expression in 60 dpf MT- treated testes (40MT) overlapped with 60 dpf testes (60CT) and 60 dpf ovaries (60CO). **d** Venn diagram of numbers of genes which showed downregulated expression in 60 dpf MT-treated testes (40MT) overlapped with 60 dpf testes (60CT) and 60 dpf ovaries (60CO)

Developing zebrafish testes and ovaries showed striking differences in the numbers of differentially expressed transcripts at 40 dpf and 60 dpf. At 40 dpf, over 5000 transcripts were differentially expressed (≥ 2-fold, FDR-adjusted *p*-value ≤0.05) between testes and ovaries (Fig. 4a and b). Of these, half were preferentially expressed in testes and half were expressed more highly in ovaries. Significantly greater numbers of sexually dimorphic transcripts were identified at 60 dpf than at 40 dpf. At 60 dpf, the number of sex differentially expressed transcripts had risen to almost 8500 (Fig. 4c and d). Similar to what was observed at 40 dpf, the proportion of testis-enriched transcripts and ovary-enriched transcripts were approximately the same at 60 dpf.

### Characterisation of the testicular differentiation programme

Transcripts with pro-male roles in fishes (*dmrt1*, *amh*, *gsdf* and *sox9a*) and genes encoding steroidogenic enzymes for 11-oxygenated androgen production (*cyp11c1*, *cyp17a1*, *hsd11b2*, *nr5a1a* and *star*) and androgen receptivity (*ar*) were more highly expressed in control testes than control ovaries during gonadal transformation and testicular differentiation (Table 3, Additional file 4). We also found upregulation of genes involved in gamma-aminobutyric acid (*gabrr2b* and *glsb*) and dopamine (*drd2l*) signalling, immune response (*il1b*, *mif* and *irf9*) and encoding histone variants (*h1f0*, *h1fx*, *h2afy2*, *histh1l* and *hist2h2l*) in control testes (CT) compared to control ovaries (CO) (Table 3, Additional file 4). The gene names and IDs can be found in Additional file 5.

**Table 3:** Expression profiles of genes involved in testicular differentiation and spermatogenesis. 40CO, 40 dpf control ovaries. 40CT, 40 dpf control testes. 60CO, 60 dpf control ovaries. 60CT, 60 dpf control testes

No.	Gene name	Gene ID	Fold change for control gonads	
Testis-Enriched Genes			40CT/40CO	60CT/60CO
Male sex determination				
1	*amh*	NM_001007779	129.92	72.07
2	*dmrt1*	NM_205628	13.89	23.43
3	*gsdf*	NM_001114668	141.09	69.01
Sertoli cell differentiation				
4	*gata4*	NM_131236	4.31	n.s.
5	*sox9a*	NM_131643	4.27	3.56
Leydig cell differentiation				
6	*pdgfra*	NM_131459	2.81	n.s.
Steroidogenesis				
7	*ar*	NM_001083123	3.38	n.s.
8	*cyp11c1*	NM_001080204	40.77	61.48
9	*cyp17a1*	NM_212806	4.84	2.63
10	*esr2b*	NM_174862	11.38	7.92
11	*hsd11b2*	NM_212720	32.13	19.24
12	*nr5a1a*	NM_131794	5.17	4.05
13	*star*	NM_131663	70.58	117.91
GABA and dopamine signalling				
14	*gabrr2b*	XM_692394.7	8.25	23.26
15	*drd2l*	NM_197935.1	n.s.	2.46
Spermiogenesis				
16	*klhl10a*	XM_002665116	n.s.	16.97
17	*odf3b*	NM_199958	n.s.	16.38
18	*tekt1*	NM_001007397	25.00	177.52
Septin signalling				
19	*sept3*	NM_001024418	n.s.	6.14
20	*sept8b*	NM_001083566	n.s.	8.13
Histone variants				
21	*h1f0*	NM_199552.1	61.83	258.58
22	*h1fx*	NM_199276.1	16.43	25.91
23	*h2afy2*	NM_001025502.1	14.10	21.92
24	*histh1l*	NM_001017660.2	10.67	10.43
25	*hist2h2l*	NM_200117.1	5.78	7.30
Immune response				
26	*mif*	NM_001043321.1	2.32	2.42
27	*il1b*	NM_212844.2	2.38	4.23
28	*irf9*	NM_205710.2	4.29	4.34
Others				
29	*dkk3b*	NM_001089545	9.03	2.67
30	*tp53*	NM_001271820	2.55	n.s.

There were developmental stage-specific differences in expression levels of testis-biased transcripts (Table 3). *G* *ata4, pdgfra* and *tp53* expression in testes were significantly higher in testes than ovaries at 40 dpf (early testicular development) but not 60 dpf (Table 3, Additional file 4). The magnitude of testis-biased expression of several pro-male genes (*dmrt1*, *gsdf*, *wt1a*, *nr0b1* and *amh*) was significantly higher at 40 dpf than 60 dpf (Table 3﻿, Additional file 4). Genes encoding sperm tail proteins *septin3*, *septin8b* and *odf3b* had been upregulated in testes compared to ovaries at 60 dpf but not 40 dpf (Table 3, Additional file 4, Additional file 6 ).

### Characterisation of the ovarian differentiation program

Transcripts implicated in teleost fish female sex determination, ovarian development, oogenesis and folliculogenesis (*foxl2*, *cyp19a1a, lhx8a*, *figla*, *bmp15*, *gdf9*, *zp21.* and *zp3b*) were more highly expressed in control ovaries than control testes (Table 4, Additional file 4). The gene names and IDs can be found in Additional file 5.

**Table 4:** Expression profiles of genes involved in ovarian differentiation and folliculogenesis. 40CO, 40 dpf control ovaries. 40CT, 40 dpf control testes. 60CO, 60 dpf control ovaries. 60CT, 60 dpf control testes

No.	Gene name	Gene ID	Fold change for control gonads	
Ovary-enriched genes			40CT/40CO	60CT/60CO
Female sex determination				
1	*foxL2a*	NM_001045252	n.s.	−3.11
2	*foxL2b*	NP_001304690	n.s.	−2.72
Folliculogenesis				
3	*bmp15*	NM_001020484	−13.76	−19.85
4	*figla*	NM_198919	−17.47	−18.88
5	*gdf9*	NM_001012383	−11.26	−14.52
6	*lhx8a*	NM_001003980	−7.81	−13.17
Zona pellucida proteins				
7	*zp2.1*	BC124100	−13.63	−27.82
8	*zp3b*	NM_131696	−16.28	−25.36
Steroid hormone and prostaglandin signalling				
9	*cyp11a1*	NM_152953	−10.79	−15.81
10	*cyp19a1a*	NM_131154	n.s.	−3.23
11	*esr2a*	NM_180966	n.s.	−2.82
Vitellogenesis				
12	*vtg1*	NM_001044897.3	n.s.	*﻿−2.50﻿*
Granulosa cell function				
13	*sox11b*	NM_131337	−6.21	−11.95
Wnt signalling				
14	*ctnnb1*	NM_131059	n.s	n.s
15	*ctnnbip1*	NM_131594	n.s	n.s
16	*lef1*	NM_131426	−3.14	−6.29
17	*tcf7*	NM_001012389	n.s	−2.66
18	*rspo1*	NM_001002352	n.s	n.s
19	*wnt4a*	NM_001040387	n.s	n.s
20	*wnt11*	NM_001144804	−3.05	−2.14
Histone variants				
21	*h1m*	NM_183071.2	−14.28	−29.14
22	*h2afx*	NM_201073.1	−6.09	−8.17
Histone modification and DNA methylation				
23	*ehmt1a*	NM_001030131.2	−3.63	−6.24
24	*ehmt2*	NM_001113615.1	n.s.	−2.13
25	*dnmt1*	NM_131189.2	−5.34	−5.02

We observed stage-dependent differences in ovarian expression patterns. Genes involved in fish folliculogenesis (*bmp15, figla, gdf9*, *lhx8a, zp2.1*, *zp3b* and *vldr*) had been more highly expressed in ovaries than testes from 40 dpf (Table 4). However, the expression levels of several pro-female transcripts (*foxl2*, *cyp19a1a*, *esr2a* and *vtg1*) had only been significantly upregulated in ovaries compared to testes at 60 dpf (Table 4). We also found higher expression of several genes involved in histone modification and DNA methylation (*ehmt1a*, *ehmt2* and *dnmt1*) and encoding histone variants (*h2afx* and *h1m*) in ovaries compared to testes (Table 4).

The expression patterns of a few transcripts with established roles in female sex determination and sex differentiation (*sox9b*, *ctnnb1*, *ctnnbip1*, *wnt4a* and *rspo1*) were not identified as sexually dimorphic in this study (Additional file 4).

### Methyltestosterone induces masculinisation and de-feminisation of the gonadal transcriptome

Substantially fewer transcripts were differentially expressed between MT-treated testes and control testes in comparison with control ovaries, particularly at 60 dpf (Table 5). Although more than five thousand transcripts were differentially expressed in MT-treated gonads compared to control ovaries at 40 dpf (Table 5), only about a thousand differentially expressed transcripts separated the transcriptomes of MT-treated gonads from control testes transcriptomes (Table 5). The transcripts differentially expressed in 40 dpf MT-treated testes compared to control ovaries overlapped considerably with those differentially expressed between control testes and control ovaries (Fig. 4a and b).

**Table 5:** Identification of genes differentially expressed (≥ 2-fold, FDR-adjusted *p*-value ≤0.05) in the gonads in response to methyltestosterone treatment at 40 dpf and 60 dpf using Baggerley’s test [83]. CO, control ovaries. CT, control testes. MT, MT-treated testes

		Experimental variable			Differentially expressed		
Stage	Experiment	Testis	Ovary	MT	Total	Up	Down
40 dpf	CO vs MT	X	X	X	5237	2415	2822
40 dpf	CT vs MT	X		X	1222	284	938
60 dpf	CO vs MT	X	X	X	7513	3433	4080
60 dpf	CT vs MT	X		X	20	9	11

At 60 dpf, the number of transcripts differentially expressed in MT-treated testes relative to control ovaries rose to seven and a half thousand (Table 5). At the same time, the number of transcripts differentially expressed between the MT-treated gonads and control testes fell to 20 (Table 5).

Marked changes indicating masculinisation and de-feminisation of the gonad transcriptome were observed in testes treated with MT to 40 dpf and 60 dpf. Among the two and a half thousand transcripts with upregulated expression in 40 dpf MT-treated testes relative to 40 dpf control ovaries (Table 5), 61% were more highly expressed in control testes than control ovaries i.e., testis-enriched (Fig. 4a). Many of these transcripts have established roles in vertebrate testicular development and spermatogenesis (Table 6, Additional file 7).

**Table 6:** Selection of genes which showed upregulated expression in MT-treated testes (MT) compared to ovaries (CO). 40CO, 40 dpf control ovaries. 40MT, 40 dpf MT-treated testes. 60CO, 60 dpf control ovaries. 60MT, 60 dpf MT-treated testes

No.	Gene name	Gene ID	Fold change for control gonads	
Testis-Enriched Genes			40MT/40CO	60MT/60CO
Male sex determination				
1	*amh*	NM_001007779	66.56	74.09
2	*dmrt1*	NM_205628	10	27.38
3	*gsdf*	NM_001114668	115.21	120.57
Sertoli cell differentiation				
4	*gata4*	NM_131236	n.s.	n.s.
5	*sox9a*	NM_131643	n.s.	n.s.
Leydig cell differentiation				
6	*pdgfra*	NM_131459	n.s.	n.s.
Steroidogenesis				
7	*ar*	NM_001083123	n.s.	n.s.
8	*cyp11c1*	NM_001080204	8.8	43.34
9	*cyp17a1*	NM_212806	n.s.	n.s.
10	*esr2b*	NM_174862	7.25	7.33
11	*hsd11b2*	NM_212720	7.40	16.49
12	*nr5a1a*	NM_131794	n.s.	n.s.
13	*star*	NM_131663	22.17	83.7
GABA and dopamine signalling				
14	*gabrr2b*	XM_692394.7	12.43	25.29
15	*drd2l*	NM_197935.1	n.s.	2.69
Spermiogenesis				
16	*klhl10a*	XM_002665116	29.96	31.04
17	*odf3b*	NM_199958	14.52	34.00
18	*tekt1*	NM_001007397	296.47	385.53
Septin signalling				
19	*sept3*	NM_001024418	5.89	6.57
20	*sept8b*	NM_001083566	11.92	11.60
Histone variants				
21	*h1f0*	NM_199552.1	322.24	493.16
22	*h1fx*	NM_199276.1	18.18	32.26
23	*h2afy2*	NM_001025502.1	12.11	26.18
24	*histh1l*	NM_001017660.2	9.44	12.75
25	*hist2h2l*	NM_200117.1	5.24	7.68
Immune response				
26	*mif*	NM_001043321.1	n.s.	2.15
27	*il1b*	NM_212844.2	n.s.	5.03
28	*irf9*	NM_205710.2	n.s.	5.51
Others				
29	*dkk3b*	NM_001089545	4.89	4.91
30	*tp53*	NM_001271820	n.s.	n.s.

Of nearly three thousand transcripts showing downregulated expression in 40 dpf MT-treated testes compared to 40 dpf control ovaries (Table 4), 74% were more highly expressed in control ovaries than control testes i.e., ovary-enriched (Fig. 4b). Genes previously implicated in fish folliculogenesis were included among the transcripts more highly expressed in ovaries compared to MT-treated testes (Table 7, Additional file 7). The proportions of transcripts with testis-enriched and ovary-enriched expression in 60 dpf MT-treated testes rose to 92% of the three and a half thousand upregulated transcripts (Fig. 4c and Table 5) and 87% of the four thousand downregulated transcripts (Fig. 4d and Table 5), respectively. This confirms that the correlation between the expression profiles of MT-treated gonads and control testes increased with developmental stage.

**Table 7:** Selection of genes which showed downregulated expression in MT-treated testes (MT) compared to ovaries (CO). 40CO, 40 dpf control ovaries. 40MT, 40 dpf MT-treated testes. 60CO, 60 dpf control ovaries. 60MT, 60 dpf MT-treated testes

No.	Gene name	Gene ID	Fold change for control gonads	
Ovary-enriched genes			40MT/40CO	60MT/60CO
Female sex determination				
1	*foxL2a*	NM_001045252	n.s.	n.s.
2	*foxL2b*	NP_001304690	n.s.	n.s.
Folliculogenesis				
3	*bmp15*	NM_001020484	−8.35	−26.10
4	*figla*	NM_198919	−7.76	−20.07
5	*gdf9*	NM_001012383	−7.13	−18.24
6	*lhx8a*	NM_001003980	−3.74	−18.32
Zona pellucida proteins				
7	*zp2.1*	BC124100	−5.71	−33.66
8	*zp3b*	NM_131696	−6.27	−37.89
Steroid hormone				
9	*cyp11a1*	NM_152953	−4.68	−15.93
10	*cyp19a1a*	NM_131154	n.s.	n.s.
11	*esr2a*	NM_180966	−3.00	−3.57
Vitellogenesis				
12	*vtg1*	NM_001044897.3	n.s.	n.s.
Granulosa cell function				
113	*sox11b*	NM_131337	−5.33	−12.80
Wnt signalling				
14	*ctnnb1*	NM_131059	n.s	n.s
15	*ctnnbip1*	NM_131594	n.s	−2.09
16	*lef1*	NM_131426	−3.01	−6.31
17	*tcf7*	NM_001012389	n.s	n.s
18	*rspo1*	NM_001002352	n.s	n.s
19	*wnt4a*	NM_001040387	n.s	n.s
20	*wnt11*	NM_001144804	n.s	−2.26
Histone variants				
21	*h1 m*	NM_183071.2	−5.40	−43.53
22	*h2afx*	NM_201073.1	−4.04	−7.85
Histone modification and DNA methylation				
23	*ehmt1a*	NM_001030131.2	−4.58	−7.73
24	*ehmt2*	NM_001113615.1	−2.81	−2.49
25	*dnmt1*	NM_131189.2	−4.32	−6.46

### Methyltestosterone induces acceleration of spermatogenesis

**Fig. 5:**
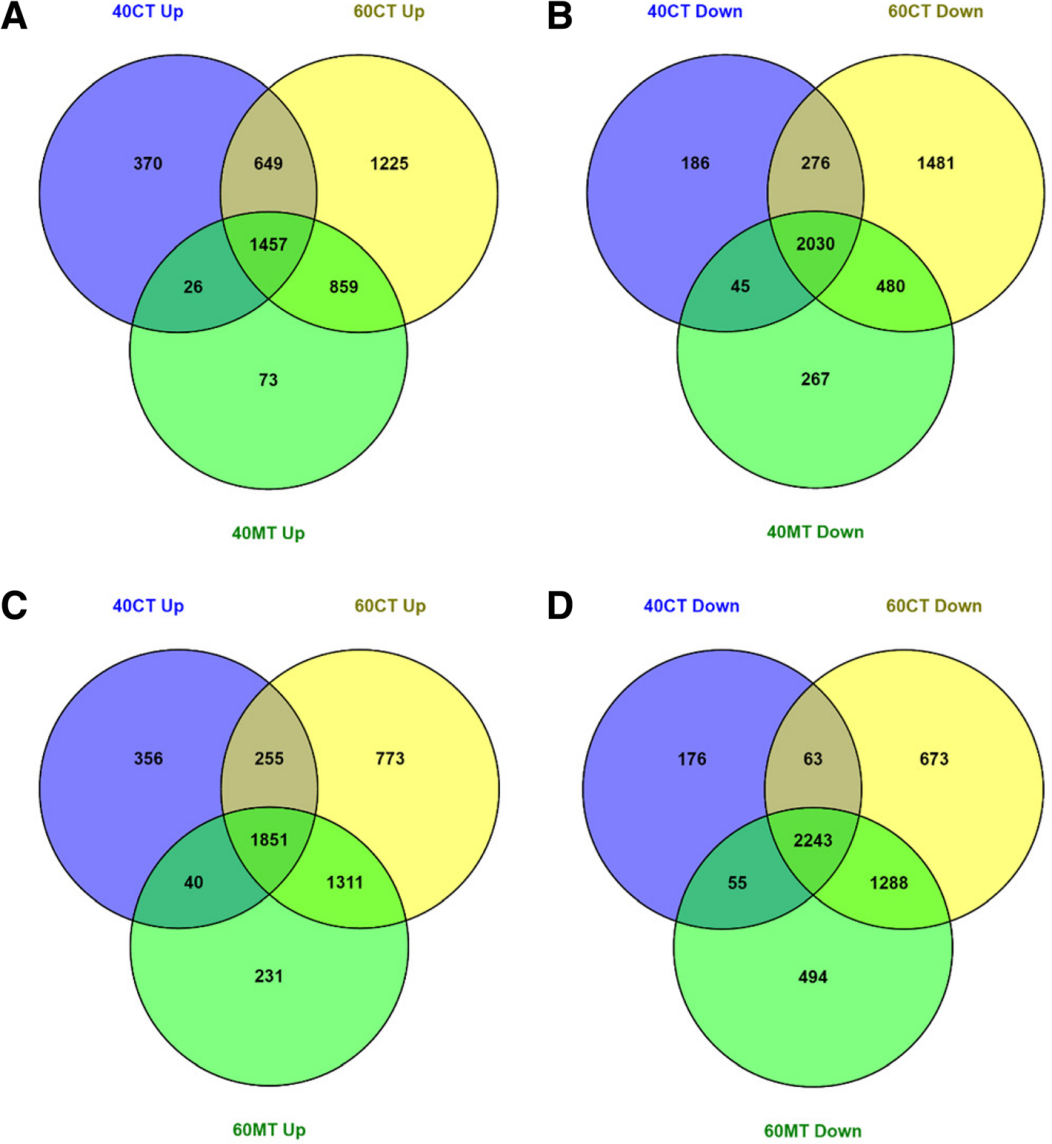
Comparison of differentially expressed genes in MT-treated zebrafish testes with testis-enriched genes of 40 dpf and 60 dpf control testes. *n* = 3 pools of 10 individuals each with the exception of 40MT which comprised of 2 pools. **a** Venn diagram of numbers of genes which showed upregulated expression in 40 dpf MT-treated testes (40MT) overlapped with numbers of genes upregulated in 40 dpf testes (40CT) and 60 dpf testes (60CT). **b** Venn diagram of numbers of genes which showed downregulated expression in 40 dpf MT-treated testes (40MT) overlapped with numbers of genes downregulated in 40 dpf testes (40CT) and 60 dpf testes (60CT). **c** Venn diagram of numbers of genes which showed upregulated expression in 60 dpf MT-treated testes (60MT) overlapped with numbers of genes upregulated in 40 dpf testes (40CT) and 60 dpf testes (60CT). **d** Venn diagram of numbers of genes which showed downregulated expression in 60 dpf MT-treated testes (60MT) overlapped with numbers of genes downregulated in 40 dpf testes (40CT) and 60 dpf testes (60CT)

The overlap between the testis-biased transcripts and transcripts showing upregulated expression in MT-treated testes relative to ovaries at 40 dpf was higher for 60 dpf control testes (96%) than 40 dpf control testes (61%) (Fig. 5a). A similar pattern was observed for the transcripts which exhibited downregulated expression in 40 dpf MT-treated testes. A greater proportion of transcripts exhibiting downregulated expression in 40 dpf MT-treated testes was shared with 60 dpf control testes (89%) than 40 dpf control testes (74%) (Fig. 5b). A comparison between the transcriptomes of 40 dpf MT-treated testes and 60 dpf control testes revealed a difference of just 136 differentially expressed transcripts (Additional file 8).

The pattern continued at 60 dpf where 92% of transcripts exhibiting upregulated expression in 60 dpf MT-treated testes overlapped with 60 dpf testis-biased transcripts compared to 55% for 40 dpf testis-biased transcripts (Fig. 5c). 87% of transcripts with downregulated expression in 60 dpf MT-treated testes compared to 60 dpf control ovaries was shared with 60 dpf control testis as compared to 56% for 40 dpf control testis (Fig. 5d).

The minor differences in the genetic cascades of 40 dpf MT-treated testes and 60 dpf control testes concurs with the gonad histological data which suggested that 17α-methyltestosterone had accelerated testicular development (Fig. 2 and Fig. 3).

### Methyltestosterone switches gonad transcription to the male program

The pathways enriched with genes exhibiting sexually dimorphic expression in zebrafish testes and ovaries were markedly different (Additional file 9). Similarly, the pathways overrepresented in MT-treated testes were considerably different from those in ovaries (Additional file 9). There was, however, substantial overlap between the pathways overrepresented in testes and MT-masculinized gonads, particularly at 60 dpf (Additional file 9).

‘Cell cycle’, ‘reproduction - progesterone-mediated oocyte maturation’ and ‘transcription’ pathways were highly enriched in zebrafish ovaries during early ovarian development (40 dpf) (Additional file 9). Zebrafish testes showed an enrichment of genes involved in ‘cytoskeleton remodelling’, and ‘immune response’, 'TGFβ signalling' and 'G-protein signalling' pathways at 40 dpf (Additional file 9).

Genes related to ‘cell cycle’, ‘reproduction - progesterone-mediated oocyte maturation’, ‘development-WNT signalling pathway’ and ‘protein folding and maturation_Angiotensin system maturation’ pathways were highly overrepresented in 60 dpf ovaries (Additional file 9). A significant overrepresentation of genes required for ‘cytoskeleton remodelling’ and ‘G-protein signalling’ were observed in 60 dpf testes (Additional file 9).

While 40 dpf zebrafish ovaries were highly enriched in genes for ‘cell cycle’, ‘development’ and ‘transcription’ pathways, MT-treated testes were enriched in genes involved in ‘immune response’, ‘cytoskeleton remodelling’ and different ‘cell cycle’ pathways (Additional file 9).

At 60 dpf, canonical 'Wnt signalling', 'integrin-mediated cell adhesion and migration', 'progesterone-mediated oocyte maturation' pathways were highly overrepresented in ovaries (Additional file 9). 60 dpf MT-treated testes were significantly enriched in ‘oxidative phosphorylation’, ‘immune response’ and ‘cell cycle’ pathways (Additional file 9).

Mitotic ‘cell cycle’ and nucleotide metabolism pathways were overrepresented in 40 dpf MT-treated testes (Additional file 9). In contrast, there was significant overrepresentation of pathways involved in ﻿'apoptosis' , Brca1 mediated regulation of transcription, the synthesis of cortisone, cortisol and androgen signalling in 40 dpf control testes (Additional file 9).

Although 60 dpf testes were highly enriched in ﻿'DNA replication', ﻿'relaxin signalling', nucleotide metabolism and ﻿'fatty acid biosynthesis' pathways (Additional file 9), no pathways were overrepresented in 60 dpf MT-treated testes.

To validate the RNA-Seq results, qPCR was conducted on 12 differentially expressed genes from four pairwise comparisons based on sex and treatment: 40CT vs. 40CO, 40MT vs. 40CO, 60CT vs. 60CO and 60MT vs. 60CO (Additional file 10). The direction of fold-change for the two platforms agreed for most of the genes (10/12 genes tested) (Additional file 10) confirming the accuracy of the RNA-Seq data.

## Discussion

In this study, we investigated the effects of MT treatment on zebrafish gonadal transcriptomes during gonadal differentiation. We found that MT treatment masculinises gonadal transcriptomes and is accompanied by morphological changes consistent with male gonad development. MT activated pro-male gene expression (*dmrt1*, *amh* and *gsdf*) and induced steroidogenic enzymes required for production of 11-oxygenated androgens (*cyp11c1* and *hsd11b2*). MT treatment also repressed gonadal expression of pro-female genes *cyp19a1a, foxl2, bmp15 and gdf9,* particularly during folliculogenesis at 60 dpf. Taken together, our data suggest that MT-induced masculinisation involves both activation of androgen receptor-mediated pathways and inhibition of aromatase.

### MT activates pro-male gene expression

We found strong upregulation of *dmrt1* expression in both control and MT-treated testes in this study, consistent with its essential role in zebrafish testis development [90]. In tilapia, upregulation of *dmrt1* expression is associated with androgen-induced gonadal masculinisation [91]. Similarly, *dmrt1* expression increased following MT treatment of XX medaka [92], and upon temperature-dependent induction of gonadal masculinisation in European seabass [93], tilapia [94], medaka [95] and pejerrey [96]. We suggest that the activation of *dmrt1* may be one of the key steps underpinning hormonal and environmental gonadal masculinisation in fish.

MT treatment increased *amh* expression in zebrafish gonads. *Amh* operates downstream of *dmrt1* in zebrafish [90] and is highly expressed in zebrafish transforming testes where it may inhibit *cyp19a1a* expression [13, 97]. Defective amh/amhr signalling in medaka causes excessive germ cell proliferation and male-to-female sex reversal in half of XY *hotei* mutants [98, 99]. The data are collectively consistent with the idea that amh signalling operates downstream of MT to repress female patterns of germ cell proliferation in teleosts.

MT treatment upregulated *gsdf* gonadal expression in zebrafish, consistent with a recent study in medaka demonstrating that MT induced *gsdf* expression in gonads [92]. In medaka, gsdf is involved in early testicular differentiation and is a key regulator of male-specific gene expression [100–103]. *Gsdf* expression has not been characterised in zebrafish. Our results suggest *gsdf* may be important for zebrafish testicular differentiation.

Interestingly, we found higher expression of *gabrr2b* and *drd2l* in control and MT-treated testes than control ovaries. Gamma-aminobutyric acid (GABA) and dopamine are important regulatory neurotransmitters in the hypothalamus-pituitary-gonadal axis. In teleost fish, both GABA and dopamine modulate production of hypothalamic gonadotrophin releasing hormone and pituitary gonadotrophins [104–110].

GABA A receptor has been implicated in Leydig cell proliferation in mice [111, 112]. GABA may regulate androgen production in rodent testes [113–115]. Dopamine type 2 receptor has been reported in mammalian testes and male germ cells where it regulates sperm capacitation and motility [116, 117]. Although dopamine antagonists are used with gonadotrophin analogues (eg. Ovaprim) for induced spawning in aquaculture [118, 119], the role of dopamine signalling in fish spermatogenesis has not been studied. Our expression data suggests that GABAergic and dopamine signalling may be important for teleost testicular development.

Genes encoding the cytokines *mif* and *il1b* had been upregulated in control and MT-testes compared to control ovaries. GO pathways involved in immune response pathways had been significantly upregulated in control testes and MT-treated testes compared to control ovaries. MIF-JAB1 and interleukin 1 signalling pathways were significantly over-represented in testes. MIF and IL-1β are cytokines involved in innate immunity and inflammation. Both act as paracrine factors responsible for regulation of testosterone production by Leydig cells in rat testes [120–124]. MIF is produced by Leydig cells and Sertoli cells where it is involved in the cross-talk between Leydig cells and testicular seminiferous tubule somatic cells, spermagonial cell migration and may be involved in regulation of sex hormone production and spermiogenesis [125–128]. IL-1β is expressed in mice testicular germ and somatic cells [129]. Interleukin 1 is important for Sertoli cell proliferation [130, 131]. Our data suggests that *mif* and *il1b* may mediate androgen-induced sex reversal in zebrafish.

Unexpectedly, we found that a few *irf* genes which included *irf9* were more highly expressed in control and MT-treated testes than control ovaries. A truncated form of *irf9* was identified as the master sex determining gene in rainbow trout [132]. Our expression data supports Yano et al. 2014’s hypothesis that interferon signalling may be involved in teleost testicular development and spermatogenesis. It may be worthwhile to study possible roles of interferon in teleost testes.

In this study, MT upregulated gene expression for several histone variants (*h1f0, h1fx, h2afy2, histh1l* and *hist2h2l*) compared to ovaries. Interestingly, gene expression of these histones had also been significantly upregulated during heat-induced masculinisation of zebrafish [61]. Testis-specific histone variants eg. histone HI variant H1t [133], histone H2B variant TSH2B [134, 135], histone H3 variant H3t [136, 137], histone H1 like [138] have been identified in mammals. Some of the testis-specific histone variants have been implicated in mammalian spermatogenesis [134, 136, 138, 139]. Chromatin compaction during spermiogenesis requires epigenetic modification of histones [139, 140], via mechanisms including chromatin modification via exchange of histone variants and histone modification [141].

### MT represses pro-female gene expression

MT treatment caused strong inhibition of *bmp15* expression in zebrafish gonads. Bmp15 is necessary for activation of *cyp19a1a* expression and therefore estrogen production in granulosa cells surrounding Stage II oocytes [142]. Loss of *cyp19a1a* expression in juvenile zebrafish ovaries leads to germ cell apoptosis and female-to-male sex reversal [70]. Our results suggest that MT treatment may suppress ovarian development by preventing bmp15-mediated *cyp19a1a* expression in granulosa cells. MT treatment also strongly inhibited genes involved in Wnt signalling pathways. Wnt signalling is important for ovarian differentiation in zebrafish [143], medaka [144, 145] and rainbow trout [146] so its repression is consistent with masculinisation.

We found that MT downregulated expression of genes involved in chromatin histone modification and DNA methylation pathways in epigenetics (*dnmt1*, *hdac11*, *ehmt1a* and *ehmt2*) compared to control ovaries. Interestingly, *hdac11* and *ehmt2* expression had been upregulated with heat-induced masculinisation in European seabass [93]. The expression levels of the histone variants *h2afx* and *h1m* had been lower in MT-treated testes than ovaries, similar to reported female-biased expression in Olive flounder [147].

### How do sex steroid hormones cause gonadal masculinisation?

Consistent with observations during MT-induced gonadal masculinisation in tilapia [40], we found *cyp11a1*, *hsd3b1* and *cyp19a1a* transcripts were reduced when juvenile zebrafish were treated with MT. We also found that MT treatment actively promoted *cyp11c1* and *hsd11b2* expression in zebrafish gonads. Cyp11c1 works together with hsd11b2 to convert androgens into 11-oxygenated androgens. This contrasts with studies in rainbow trout which found downregulated *cyp11c1* expression during androgen-induced gonadal masculinisation using 11β-hydroxyandrostenedione [41, 45]. Despite both being 11-KT precursors, MT and 11β-hydroxyandrostenedione produce different intermediate products with different androgenic potencies. It is possible that MT induces gonadal masculinisation by different mechanisms to those employed by 11β-hydroxyandrostenedione, perhaps via these differing intermediate products.

Our results suggest that androgen production may also be important for natural juvenile ovary-to-testis gonadal transformation in zebrafish. *Cyp11c1* and *hsd11b2* expression was higher in transforming testes than juvenile ovaries during juvenile ovary-to-testis transformation at 40 dpf. This concurs with the higher levels of *cyp11c1* expression previously reported in juvenile ovotestes compared to juvenile ovaries amid zebrafish juvenile ovary-to-testis transformation [13, 143]. This indicates that the steroidogenic enzymes required for androgen production are active during early stages of juvenile ovary-to-testis transformation. We found that pathways involved in cortisol biosynthesis from cholesterol and cortisone biosynthesis and metabolism and androgen receptor nuclear signalling were higher in transforming testes than MT-treated testes at 40 dpf. Cortisol and androgen signalling pathways were shown to be linked in high temperature-induced masculinisation of pejerrey via *hsd11b2* [148]. This suggests that *hsd11b2*-mediated androgen production may be important for testicular differentiation in zebrafish.

Taken together, our data show that increased 11-oxygenated androgen production and decreased aromatase expression may be equally important for MT-induced gonadal masculinisation in zebrafish.

### MT alters germ cell proliferation rates in juvenile ovaries

The juvenile ovary-to-testis transformation process in zebrafish is characterised by oocyte apoptosis [71]. In zebrafish, genes in pro-apoptotic pathways (tp53) have been implicated in testicular differentiation [149], while anti-apoptotic pathways (NF-κB) promote ovarian differentiation [150]. Partial or complete germ cell depletion in gonads invariably leads to gonadal masculinisation [88, 149, 151, 152].

Gonadal hormones can regulate germ cell apoptosis in vertebrates [153, 154] including teleost fish [5]. In zebrafish, 17α-ethinylestradiol and fadrozole can alter germ cell apoptosis and proliferation during gonad differentiation [54]. Several genes that regulate cell apoptosis and proliferation are also involved in gonadal sexual differentiation [155]. A threshold of germ cells or meiotic oocytes is required for ovarian development in zebrafish [152, 156] such that the phenotypic sex in zebrafish depends critically on the number of germ cells in gonads during early development [88, 152, 157]. How germ cell numbers maintain female identity is not fully understood.

We observed that MT treatment downregulates genes involved in DNA replication in early S phase, Estrogen Receptor 1 (Esr1) regulation of G1/S phase, mitogenic action of Estradiol /Esr1 and ligand-dependent activation of the Estrogen receptor 1/ Specificity Protein (Esr1/Sp) pathway in 40 dpf juvenile ovaries. We suggest that androgens may induce female-to-male sex reversal via modulation of germ cell proliferation rates. MT-induced repression of estrogen-activated mitotic pathways in juvenile ovaries leads us to hypothesise that MT may trigger female-to-male sex reversal via inhibition of estrogen-activated proliferation of oocytes in zebrafish ovaries. This would reduce oocyte numbers below the threshold, leading to activation of male-specific gene expression in gonadal somatic cells.

In this study, we used transgenic zebrafish derived from domesticated zebrafish strains. Domesticated zebrafish strains use polygenic sex determination [58, 59] and are sensitive to environmental and hormonal factors [32, 53, 61, 63, 158]. The sensitivity of domesticated zebrafish strains to hormonal effects on gonad development render them good models for determining the mode of action of androgens on induction of testicular differentiation. Evidence comparing Atlantic Silverside utilising genetic sex determination (GSD) and temperature sex determination (TSD) suggest that expression of *cyp19a1a*, a key gene in ovarian development, was earlier, stronger, more sexually dimorphic and less temperature sensitive in GSD strains than TSD strains [159]. It is possible that the thresholds of environmental and hormonal sensitivity for key sex determining loci may vary between wild (ZZ/ZW sex determination) and domesticated (polygenic sex determination) zebrafish strains. Different mechanisms may be activated for androgen-induced sex reversal of wild zebrafish populations. Future studies using wild strains would facilitate elucidation of the pathways involved. Due to the polygenic mode of sex determination and lack of sex-linked markers in domesticated zebrafish strains, we were unable to distinguish between male and female zebrafish prior to morphological gonadal differentiation. Therefore, the MT treatment was conducted on zebrafish with unknown proportions of male and female sexual genotypes. This resulted in production of populations comprising males and neo-males. The development and use of all-male and all-female zebrafish lines [160] in future studies would help further clarify whether MT treatment has different effects on genetic males and females.

## Conclusion

In conclusion, zebrafish MT-induced gonadal masculinisation involves activation of pro-male gene expression and repression of pro-female gene expression. Steroidogenic enzymes critical for 11-oxygenated androgen production (*cyp11c1* and *hsd11b2*) and estrogen production (*cyp19a1a*) were among genes differentially expressed in response to MT treatment suggesting that androgen production and aromatase inhibition appear to both be important for zebrafish androgen-induced gonadal masculinisation. MT-induced epigenetic modification of histones and inhibition of estrogen-activated germ cell proliferation via *cyp19a1a* downregulation may be two of the key mechanisms which mediate female-to-male sex reversal in zebrafish.

## Additional files

Additional file 1: Detailed methods for qRT-PCR validation of RNA-Seq data. (DOCX 20kb)

Additional file 2: Survival rates and sex ratios of control and MT-treated zebrafish. (DOCX 14kb)

Additional file 3: Read counts, percentage of reads mapped and RPKM. (DOCX 16kb)

Additional file 4: Genes differentially expressed by 2-fold or more in control testes and ovaries. (XLS 1933kb)

Additional file 5: ﻿Gene IDs, gene symbols and gene names. (DOCX 18kb)

Additional file 6: Genes differentially expressed by 2-fold or more in 40 dpf testes compared to 60 dpf testes. (XLS 230kb)

Additional file 7:﻿ Genes differentially expressed by 2-fold or more in MT-treated testes compared to ovaries. (XLS 3834kb)

Additional file 8: Genes differentially expressed by 2-fold or more in 40 dpf MT-treated testes compared to 60 dpf testes. (XLS 40kb)

Additional file 9: Top ten GeneGo pathways significantly enriched in pairwise comparisons of sex (testes vs. ovaries), treatment (control vs. MT-treated) and time point (40 dpf vs. 60 dpf). (DOCX 20kb)

Additional file 10: Results of qRT-PCR validation of RNA-Seq data. The RNA-seq results are expressed in terms of normalized fold-change (adjusted p-value < 0.05) while qPCR data are expressed as the average relative fold change between samples normalised against the eef1a1l1 reference gene. Fold change values are compared against the 40 dpf control ovary group (40CO) and 60 dpf control ovary group (60CO). (DOCX 17 kb)

## Abbreviations

CO: Control ovary
CT: Control testis
dpf: Days post fertilisation
Esr1: Estrogen Receptor 1
GO: Gene ontology consortium
MT: 17α-methyltestosterone
PCR: Polymerase chain reaction
qRT-PCR: Quantitative real-time polymerase chain reaction
RNA-seq: RNA sequencing
RPKM: Reads per kilobase per million mapped reads
Sp: Specificity Protein transcription factor
