# Supplementary material for: Histological and transcriptomic effects of 17α-methyltestosterone on zebrafish gonad development
Source: BMC Genomics. 2017 Jul 24;18:557. doi: 10.1186/s12864-017-3915-z (PMC5523153; doi:10.1186/s12864-017-3915-z)
Supplement: Supplementary file 1 — Detailed methods for qRT-PCR validation of RNA-Seq data. (DOCX 20kb) [file 12864_2017_3915_MOESM1_ESM.docx]

### Methods for validation of differentially expressed genes using quantitative RT-PCR

Validation was conducted using quantitative real-time PCR (qRT-PCR) on a selection of 12 differentially expressed transcripts identified in the RNA-seq experiment. Four genes which were expressed more highly in ovaries than testes (*bmp15*, *gdf9*, *lhx8a*, *figlα*) and eight genes which exhibited higher expression in testes than ovaries (*amh*, *dmrt1*, *gsdf*, *wt1a*, *cyp11c1*, *hormad1*, *star*, *sycp3l*) were chosen for qPCR validation (Table 1).

Total RNA was isolated from dissected testis and ovary samples, processed and pooled as for the RNA-sequencing experiment. The gonad RNA samples comprising gonads from ten individuals per treatment group were pooled to reduce differences in expression profiles observed due to individual variation. Three biological replicates of gonad pools consisting of 10 individuals each were generated for each exposure group (methyltestosterone treated and solvent control), gonad type (testis or ovary) and developmental stage (40 dpf or 60 dpf) except for the 40 dpf methyltestosterone treated testis group, which was limited to two biological replicates. A total of seventeen gonad RNA pools were created: 40CO1-3 (40 dpf control female ovary pools 1 to 3), 40CT1-3 (40 dpf control male testis pools 1 to 3), 40MT1-2 (40 dpf MT-treated male testis pools 2 and 3), 60CO1-3 (60 dpf control female ovary pools 1 to 3), 60CT1-3 (60 dpf control male testis pools 1 to 3), and 60MT1-3 (60 dpf MT-treated male testis pools 1 to 3).

250 ng of total RNA from each pool was used as the template for cDNA synthesis. First strand cDNA synthesis was performed using Superscript III Reverse transcriptase (Invitrogen, Thermo Fisher Scientific, Waltham, Massachusetts, USA) with a 3:1 molar ratio mix of random hexamer to Oligo (dT)_20_ primers (v/v) at a final concentration of 2.5 µM (Integrated DNA Technologies, Coralville, Iowa, USA). A combination of random hexamers and Oligo (dT)_20_ primers were used to prime the cDNA synthesis to improve cDNA yield and promote the production of full-length cDNA products from mRNAs respectively.

Commercially available Taqman Gene Expression Assays (Applied Biosystems, Thermo Fisher Scientific, Waltham, Massachusetts, USA) specific to our genes of interest were used (Table 1). Primers were designed to span across intron-exon boundaries as much as possible, to minimise genomic DNA amplification. *Eef1a1l1* were used as the internal control for normalisation of all qPCR experiments.

Three biological replicates were conducted for each gene to verify the gene expression patterns observed for the RNA-seq experiment. Each TaqMan qPCR reaction was performed in triplicate. The TaqMan Gene Expression Assay (Applied Biosystems, Thermo Fisher Scientific, Waltham, Massachusetts, USA) for each gene of interest was used with the TaqMan Universal Master Mix II with UNG (Applied Biosystems, Thermo Fisher Scientific, Waltham, Massachusetts, USA) in accordance to manufacturer’s recommendations. The PCR cycling conditions used were 50°C for 2 min, 95°C for 10 min followed by 40 cycles at 95°C for 15 secs and 60°C for 1 min. Each reaction (20µl total volume) contained 10µl of TaqMan Universal Master Mix II, 1 µl of TaqMan Gene Expression Assay, 1 µl of template cDNA (1:10 diluted RT product) and MilliQ water. All real-time PCR reactions were performed on a Stratagene Mx3000p (Agilent Technologies, Santa Clara, California, USA) Real Time-PCR thermal cycler using the Comparative Quantitation program and analysed using the MxPro software (Agilent Technologies, Santa Clara, California, USA). ΔCt for each target gene was calculated using raw Ct values normalised to *eef1a1l1*. The comparative CT method (ΔΔCt) was used to determine relative gene expression. The calibrator was chosen based on the samples exhibiting the greatest expression level. Relative expression was expressed as fold change (Fold Change = 2^−ΔΔCt^). RNA-seq (RPKM values) and qPCR fold change values were compared for direction of change in expression (upregulation or downregulation) and magnitude of expression levels.

**Table 1.** **Taqman gene expression assays utilised for qPCR validation**

| # | Gene symbol | Gene description | Amplicon size (bp) | RefSeq ID | Assay ID |
| --- | --- | --- | --- | --- | --- |
| 1 | *amh* | Anti-Mullerian hormone | 109 | [NM_001007779.1](http://www.ncbi.nlm.nih.gov/nuccore/NM_001007779.1) | Dr03141032_g1 |
| 2 | *dmrt1* | Doublesex and mab-3 related transcription factor 1 | 73 | [NM_205628.1](http://www.ncbi.nlm.nih.gov/nuccore/NM_205628.1) | Dr03107393_m1 |
| 3 | *hormad1* | HORMA domain containing 1 | 108 | [NM_001002357.1](http://www.ncbi.nlm.nih.gov/nuccore/NM_001002357.1) | Dr03140416_m1 |
| 4 | *wt1a* | Wilms tumor 1a | 97 | [NM_131046.1](http://www.ncbi.nlm.nih.gov/nuccore/NM_131046.1) | Dr03093254_m1 |
| 5 | *cyp11c1* | Cytochrome P450, family 11, subfamily C, polypeptide 1 | 84 | [NM_001080204.1](http://www.ncbi.nlm.nih.gov/nuccore/NM_001080204.1) | Dr03079075_m1 |
| 6 | *gsdf* | Gonadal somatic cell derived factor | 87 | [NM_001114668.1](http://www.ncbi.nlm.nih.gov/nuccore/NM_001114668.1) | Dr03436116_m1 |
| 7 | *star* | Steroidogenic acute regulatory protein | 95 | [NM_131663.1](http://www.ncbi.nlm.nih.gov/nuccore/NM_131663.1) | Dr03112289_m1 |
| 8 | *sycp3l* | Synaptonemal complex protein 3 | 105 | [NM_001040350.1](http://www.ncbi.nlm.nih.gov/nuccore/NM_001040350.1) | Dr03091302_g1 |
| 9 | *bmp15* | Bone morphogenetic protein 15 | 143 | [NM_001020484.1](http://www.ncbi.nlm.nih.gov/nuccore/NM_001020484.1) | Dr03135417_m1 |
| 10 | *gdf9* | Growth differentiation factor 9 | 86 | NM_001012383.1 | Dr03096331_m1 |
| 11 | *lhx8a* | LIM homeobox 8a | 90 | NM_001003980.2 | Dr03076493_m1 |
| 12 | *figla* | Factor in the germline alpha | 85 | NM_198919.2 | Dr03119349_g1 |
| 13 | *eef1a1l1* | Eukaryotic translation elongation factor 1 alpha 1, like 1 | 106 | NM_131263.1 | Dr03432748_m1 |
