# Supplementary material for: Histological and transcriptomic effects of 17α-methyltestosterone on zebrafish gonad development
Source: BMC Genomics. 2017 Jul 24;18:557. doi: 10.1186/s12864-017-3915-z (PMC5523153; doi:10.1186/s12864-017-3915-z)
Supplement: Supplementary file 2 — Survival rates and sex ratios of control and MT-treated zebrafish. (DOCX 14kb) [file 12864_2017_3915_MOESM2_ESM.docx]

**Table 1. Survival rates, sex ratios and mean standard length at 40 dpf of zebrafish exposed to 100 ng/L of MT from 20 to 40 dpf. n = 20 per replicate.**

| 40 dpf | Solvent control | | | | | Methyltestosterone treatment | | |
| --- | --- | --- | --- | --- | --- | --- | --- | --- |
| Replicate | 1 | 2 | 3 | 4 | 5 | 1 | 2 | 3 |
| Survival rate | 100% | 100% | 100% | 100% | 95% | 100% | 100% | 100% |
| Sex ratio  (F to M) | 70%:30% | 75%:25% | 60%:40% | 25%:75% | 37%:63% | 5%:95% | 0%:100% | 0%:100% |
| Overall sex ratio (F to M) | 54%:46% | | | | | 1.7%:98.3% | | |
| Standard length (mm) | 10±0.9 | 9.5±1.1 | 10±0.7 | 11.3±1.3 | 11.2±1.2 | 9.7±1.2 | 11.2±0.6 | 11.8±1.1 |

**Table 2. Survival rates, sex ratios and mean standard length at 60 dpf of zebrafish exposed to 100 ng/L of MT from 20 to 60dpf. n = 20 per replicate.**

| 60 dpf | Solvent control | | | | | Methyltestosterone treatment | | | | |
| --- | --- | --- | --- | --- | --- | --- | --- | --- | --- | --- |
| Replicate | 1 | 2 | 3 | 4 | 5 | 1 | 2 | 3 | 4 | 5 |
| Survival rate | 90% | 95% | 95% | 95% | 95% | 100% | 95% | 100% | 100% | 95% |
| Sex ratio  (F to M) | 33%:67% | 74%:26% | 58%:42% | 53%:47% | 26%:76% | 0%:100% | 0%:100% | 0%:100% | 0%:100% | 0%:100% |
| Overall sex ratio (F to M) | 46%:54% | | | | | 0%:100% | | | | |
| Standard length (mm) | 13.4±1.9 | 13.6±1.1 | 14.6±1.2 | 13.3±3.2 | 14.1±1.9 | 14.0±1.2 | 13.9±0.9 | 13.3±1.1 | 14.2±1.5 | 14.9±1.6 |
