# Supplementary material for: Histological and transcriptomic effects of 17α-methyltestosterone on zebrafish gonad development
Source: BMC Genomics. 2017 Jul 24;18:557. doi: 10.1186/s12864-017-3915-z (PMC5523153; doi:10.1186/s12864-017-3915-z)
Supplement: Supplementary file 3 — Read counts, percentage of reads mapped and RPKM. (DOCX 16kb) [file 12864_2017_3915_MOESM3_ESM.docx]

**Table 1. Table of read counts, percentage of reads mapped and RPKM for 40 dpf gonads.**

| **Sample** | **Run** | **Read count** | **Total read count** | **% mapped** | **RPKM≥1** | **RPKM≥5** |
| --- | --- | --- | --- | --- | --- | --- |
| **40dpf Solvent control ovary** |  |  |  |  |  |  |
| **40CO1** | 1 | 27,753,518 | 27,753,518 | 83.45% | 12,719 | 8,118 |
| **Average** | 27,753,518 | 83.45% | 12,719 | 8,118 |  |  |
| **40CO2** | 1 | 34,723,386 | 34,723,386 | 83.58% | 12,452 | 7,900 |
| **Average** | 34,723,386 | 83.58% | 12,452 | 7,900 |  |  |
| **40CO3** | 1 | 41,026,976 | 41,026,976 | 83.10% | 12,681 | 8,122 |
| **Average** | 41,026,976 | 83.10% | 12,681 | 8,122 |  |  |
| **40 dpf Solvent control testis** |  |  |  |  |  |  |
| **40CT1** | 1 | 33,575,790 | 33,575,790 | 80.19% | 18,819 | 11,586 |
| **Average** | 33,575,790 | 80.19% | 18,819 | 11,586 |  |  |
| **40CT2** | 1 | 41,973,836 | 41,973,836 | 78.35% | 18,925 | 11,573 |
| **Average** | 41,973,836 | 78.35% | 18,925 | 11,573 |  |  |
| **40CT3** | 1 | 35,471,202 | 35,471,202 | 79.20% | 18,974 | 11,918 |
| **Average** | 35,471,202 | 79.20% | 18,974 | 11,918 |  |  |
| **40 dpf Methyltestosterone testis** |  |  |  |  |  |  |
| **40MT1** | 1 | 11,203,340 | 33,648,924 | 77.23% | 18,430 | 11,550 |
| **Average** | 11,203,340 | 77.23% | 18,430 | 11,550 |  |  |
| **40MT2** | 1 | 10,017,654 | 29,828,654 | 77.44% | 18,192 | 11,427 |
| **Average** | 10,017,654 | 77.44% | 18,192 | 11,427 |  |  |
| **Total** | 278,002,286 |  |  |  |  |  |

**Table 2. Table of read counts, percentage of reads mapped and RPKM for 60 dpf gonads.**

| **Sample** | **Run** | **Read count** | **Total read count** | **% mapped** | **RPKM≥1** | **RPKM≥5** |
| --- | --- | --- | --- | --- | --- | --- |
| **60dpf Solvent control ovary** |  |  |  |  |  |  |
| **60CO1** | 1 | 14,818,458 | 50,417,800 | 84.06% | 12,205 | 8,128 |
| **60CO1** | 2 | 17,984,364 | 84.18% | 12,250 | 8,161 |  |
| **60CO1** | 3 | 17,614,978 | 84.18% | 12,273 | 8,132 |  |
| **Average** | 16,805,933 | 84.14% | 12,243 | 8,140 |  |  |
| **60CO2** | 1 | 13,012,626 | 42,857,246 | 83.81% | 12,354 | 8,250 |
| **60CO2** | 2 | 14,759,230 | 83.96% | 12,366 | 8,245 |  |
| **60CO2** | 3 | 15,085,390 | 83.94% | 12,322 | 8,299 |  |
| **Average** | 14,285,749 | 83.90% | 12,347 | 8,265 |  |  |
| **60CO3** | 1 | 16,491,146 | 55,777,440 | 83.53% | 12,223 | 8,193 |
| **60CO3** | 2 | 19,803,674 | 83.66% | 12,233 | 8,170 |  |
| **60CO3** | 3 | 19,482,620 | 83.68% | 12,193 | 8,145 |  |
| **Average** | 18,592,480 | 83.62% | 12,216 | 8,169 |  |  |
| **60dpf Solvent control testis** |  |  |  |  |  |  |
| **60CT1** | 1 | 12,782,706 | 41,491,450 | 77.54% | 19,026 | 11,923 |
| **60CT1** | 2 | 14,389,824 | 77.44% | 19,006 | 11,889 |  |
| **60CT1** | 3 | 14,318,920 | 77.44% | 19,017 | 11,924 |  |
| **Average** | 13,830,483 | 77.47% | 19,016 | 11,912 |  |  |
| **60CT2** | 1 | 13,603,166 | 45,274,320 | 77.40% | 19,180 | 12,114 |
| **60CT2** | 2 | 16,161,846 | 77.49% | 19,223 | 12,073 |  |
| **60CT2** | 3 | 15,509,308 | 77.49% | 19,185 | 12,113 |  |
| **Average** | 15,091,440 | 77.46% | 19,196 | 12,100 |  |  |
| **60CT3** | 1 | 13,812,454 | 46,836,830 | 77.66% | 18,995 | 11,929 |
| **60CT3** | 2 | 16,545,056 | 77.72% | 18,900 | 11,914 |  |
| **60CT3** | 3 | 16,479,320 | 77.74% | 18,973 | 11,899 |  |
| **Average** | 15,612,277 | 77.71% | 18,956 | 11,914 |  |  |
| **60dpf Methyltestosterone testis** |  |  |  |  |  |  |
| **60MT1** | 1 | 29,615,490 | 29,615,490 | 77.08% | 19,191 | 11,872 |
| **Average** | 29,615,490 | 77.08% | 19,191 | 11,872 |  |  |
| **60MT2** | 1 | 35,097,066 | 35,097,066 | 76.87% | 18,713 | 11,686 |
| **Average** | 35,097,066 | 76.87% | 18,713 | 11,686 |  |  |
| **60MT3** | 1 | 31,742,860 | 31,742,860 | 76.93% | 19,180 | 12,038 |
| **Average** | 31,742,860 | 76.93% | 19,180 | 12,038 |  |  |
| **Total** | 379,110,502 |  |  |  |  |  |
