# Supplementary material for: Histological and transcriptomic effects of 17α-methyltestosterone on zebrafish gonad development
Source: BMC Genomics. 2017 Jul 24;18:557. doi: 10.1186/s12864-017-3915-z (PMC5523153; doi:10.1186/s12864-017-3915-z)
Supplement: Supplementary file 5 — ﻿Gene IDs, gene symbols and gene names. (DOCX 18kb) [file 12864_2017_3915_MOESM5_ESM.docx]

**Supplementary Table S3. Table of gene IDs, gene symbols and gene names (male).**

| **No.** | **Gene ID** | **Gene symbol** | **Gene name** |
| --- | --- | --- | --- |
| **1** | NM_001007779 | *amh* | *anti-Mullerian hormone* |
| **2** | NM_001083123 | *ar* | *androgen receptor* |
| **3** | NM_001080204 | *cyp11c1* | *cytochrome P450, family 11, subfamily C, polypeptide 1* |
| **4** | NM_212806 | *cyp17a1* | cytochrome P450, family 17, subfamily A, polypeptide 1 |
| **5** | NM_197935.1 | *drd2l* | *dopamine receptor D2 like* |
| **6** | NM_001089545 | *dkk3b* | *dickkopf WNT signaling pathway inhibitor 3b* |
| **7** | NM_205628 | *dmrt1* | *doublesex and mab-3 related transcription factor* |
| **8** | NM_174862 | *esr2b* | *estrogen receptor 2b* |
| **9** | NM_131236 | *gata4* | *GATA binding protein 4* |
| **10** | NM_001114668 | *gsdf* | *gonadal soma derived factor* |
| **11** | XM_692394.7 | *gabrr2b* | *gamma-aminobutyric acid (GABA) A receptor, rho 2b* |
| **12** | NM_199552.1 | *h1f0* | *H1 histone family, member 0* |
| **13** | NM_199276.1 | *h1fx* | *H1 histone family, member X* |
| **14** | NM_001025502.1 | *h2afy2* | *H2A histone family, member Y2* |
| **15** | NM_001017660.2 | *histh1l* | *histone H1 like* |
| **16** | NM_212720 | *hsd11b2* | *hydroxysteroid (11-beta) dehydrogenase 2* |
| **17** | XM_002665116 | *klhl10a* | *kelch-like family member 10a* |
| **18** | NM_212844.2 | *il1b* | *interleukin 1, beta* |
| **19** | NM_205710.2 | *irf9* | *interferon regulatory factor 9* |
| **20** | NM_001043321.1 | *mif* | *macrophage migration inhibitory factor* |
| **21** | NM_131794 | *nr5a1a* | *nuclear receptor subfamily 5, group A, member 1a* |
| **22** | NM_199958 | *odf3b* | *outer dense fiber of sperm tails 3B* |
| **23** | NM_131459 | *pdgfra* | *platelet-derived growth factor receptor a* |
| **24** | NM_001024418 | *sept3* | *septin 3* |
| **25** | NM_001083566 | *sept8b* | *septin 8b* |
| **26** | NM_131643 | *sox9a* | *SRY-box containing gene 9a* |
| **27** | NM_131663 | *star* | *steroidogenic acute regulatory protein* |
| **28** | NM_001007397 | *tekt1* | *tektin 1* |
| **30** | NM_001271820 | *tp53* | *tumor protein p53* |

**Supplementary Table S4. Table of gene IDs, gene symbols and gene names (female).**

| **No.** | **Gene ID** | **Gene symbol** | **Gene name** |
| --- | --- | --- | --- |
| **1** | NM_001020484 | *bmp15* | *bone morphogenetic protein 15* |
| **2** | NM_131059 | *ctnnb1* | *catenin (cadherin-associated protein), beta 1* |
| **3** | NM_131594 | *ctnnbip1* | *catenin, beta interacting protein 1* |
| **4** | NM_152953 | *cyp11a1* | *cytochrome P450, family 11, subfamily A, polypeptide 1* |
| **5** | NM_131154 | *cyp19a1a* | *cytochrome P450, family 19, subfamily A, polypeptide 1a* |
| **6** | NM_131189.2 | *dnmt1* | *DNA (cytosine-5-)-methyltransferase 1* |
| **7** | NM_180966 | *esr2a* | *estrogen receptor 2a* |
| **8** | NM_001030131.2 | *ehmt1a* | *euchromatic histone-lysine N-methyltransferase 1a* |
| **9** | NM_001113615.1 | *ehmt2* | *euchromatic histone-lysine N-methyltransferase 2* |
| **10** | NM_198919 | *figla* | *folliculogenesis specific bHLH transcription factor* |
| **11** | NM_001045252 | *foxL2a* | *forkhead box L2A* |
| **12** | NP_001304690 | *foxL2b* | *forkhead box L2B* |
| **13** | NM_001012383 | *gdf9* | *growth differentiation factor 9* |
| **14** | NM_183071.2 | *h1m* | *linker histone H1M* |
| **15** | NM_201073.1 | *h2afx* | *H2A histone family, member X* |
| **16** | NM_131426 | *lef1* | *lymphoid enhancer-binding factor 1* |
| **17** | NM_001003980 | *lhx8a* | *LIM homeobox 8a* |
| **17** | NM_001002352 | rspo1 | *R-spondin 1* |
| 18 | NM_131644 | *sox9b* | *SRY (sex determining region Y)-box 9b* |
| 19 | NM_131337 | *sox11b* | *SRY-Box Containing Gene 11b* |
| **20** | NM_001012389 | *tcf7* | *transcription factor 7 (T-cell specific, HMG-box)* |
| **21** | NM_001044897.3 | *vtg1* | *vitellogenin 1* |
| **22** | NM_001040387 | *wnt4a* | *wingless-type MMTV integration site family, member 4a* |
| **23** | NM_001144804 | *wnt11* | *wingless-type MMTV integration site family, member 11* |
| **24** | BC124100 | *zp2.1* | *zona pellucida glycoprotein 2, tandem duplicate 1* |
| **25** | NM_131696 | *zp3b* | *zona pellucida glycoprotein 3b* |
