# Supplementary material for: Histological and transcriptomic effects of 17α-methyltestosterone on zebrafish gonad development
Source: BMC Genomics. 2017 Jul 24;18:557. doi: 10.1186/s12864-017-3915-z (PMC5523153; doi:10.1186/s12864-017-3915-z)
Supplement: Supplementary file 9 — Top ten GeneGo pathways significantly enriched in pairwise comparisons of sex (testes vs. ovaries), treatment (control vs. MT-treated) and time point (40 dpf vs. 60 dpf). (DOCX 20kb) [file 12864_2017_3915_MOESM9_ESM.docx]

Table 1 The top ten GeneGo pathways enriched among genes significantly up-regulated in 40 dpf testes and ovaries.

| Pathways |  |
| --- | --- |
| Cell adhesion_Chemokines and adhesion | Testis-enriched |
| Cytoskeleton remodeling_TGF, WNT and cytoskeletal remodeling | Testis-enriched |
| Cytoskeleton remodeling_Cytoskeleton remodeling | Testis-enriched |
| Immune response_MIF-JAB1 signaling | Testis-enriched |
| Ovarian cancer (main signaling cascades) | Testis-enriched |
| Development_TGF-beta-dependent induction of EMT via RhoA, PI3K and ILK. | Testis-enriched |
| Development_Regulation of epithelial-to-mesenchymal transition (EMT) | Testis-enriched |
| Stimulation of TGF-beta signaling in lung cancer | Testis-enriched |
| Development_WNT signaling pathway. Part 1. Degradation of beta-catenin in the absence WNT signaling | Testis-enriched |
| G-protein signaling_Ras family GTPases in kinase cascades (schema) | Testis-enriched |
| Cell cycle_Role of APC in cell cycle regulation | Ovary-enriched |
| Cell cycle_Spindle assembly and chromosome separation | Ovary-enriched |
| Reproduction_Progesterone-mediated oocyte maturation | Ovary-enriched |
| Cell cycle_Initiation of mitosis | Ovary-enriched |
| Cell cycle_Start of DNA replication in early S phase | Ovary-enriched |
| Transcription_Role of heterochromatin protein 1 (HP1) family in transcriptional silencing | Ovary-enriched |
| N-Glycan biosynthesis p1 | Ovary-enriched |
| Galactose metabolism | Ovary-enriched |
| Delta508-CFTR traffic / ER-to-Golgi in CF | Ovary-enriched |
| wtCFTR traffic / ER-to-Golgi (normal) | Ovary-enriched |

Table 2 The top ten GeneGo pathways enriched among genes significantly up-regulated in 60 dpf testes and ovaries.

| Pathways | |
| --- | --- |
| Cell adhesion_Chemokines and adhesion | Testis-enriched |
| Cytoskeleton remodeling_TGF, WNT and cytoskeletal remodeling | Testis-enriched |
| Ovarian cancer (main signaling cascades) | Testis-enriched |
| Protein folding_Membrane trafficking and signal transduction of G-alpha (i) heterotrimeric G-protein | Testis-enriched |
| Cytoskeleton remodeling_Cytoskeleton remodeling | Testis-enriched |
| G-protein signaling_G-Protein alpha-12 signaling pathway | Testis-enriched |
| Immune response_IL-1 signaling pathway | Testis-enriched |
| G-protein signaling_Regulation of p38 and JNK signaling mediated by G-proteins | Testis-enriched |
| Development_PIP3 signaling in cardiac myocytes | Testis-enriched |
| Oxidative phosphorylation | Testis-enriched |
| Reproduction_Progesterone-mediated oocyte maturation | Ovary-enriched |
| G-protein signaling_RhoA regulation pathway | Ovary-enriched |
| Protein folding and maturation_Angiotensin system maturation \ Human version | Ovary-enriched |
| Cell cycle_Initiation of mitosis | Ovary-enriched |
| Cytoskeleton remodeling_TGF, WNT and cytoskeletal remodeling | Ovary-enriched |
| Protein folding and maturation_Angiotensin system maturation \ Rodent version | Ovary-enriched |
| Neurophysiological process_Receptor-mediated axon growth repulsion | Ovary-enriched |
| Development_WNT signaling pathway. Part 2 | Ovary-enriched |
| Cell cycle_Nucleocytoplasmic transport of CDK/Cyclins | Ovary-enriched |
| Cell adhesion_Integrin-mediated cell adhesion and migration | Ovary-enriched |

Table 3 The top ten GeneGo pathways enriched among genes significantly expressed between 40 dpf methytestosterone-treated testes and 40 dpf control ovaries.

| Pathways |  |
| --- | --- |
| Cytoskeleton remodeling_Keratin filaments | MT-testis-enriched |
| Cell adhesion_Role of tetraspanins in the integrin-mediated cell adhesion | MT-testis-enriched |
| Immune response_Antigen presentation by MHC class I | MT-testis-enriched |
| Cell cycle_Nucleocytoplasmic transport of CDK/Cyclins | MT-testis-enriched |
| Protein folding and maturation_Bradykinin / Kallidin maturation | MT-testis-enriched |
| Cell cycle_Spindle assembly and chromosome separation | MT-testis-enriched |
| Immune response_IL-1 signaling pathway | MT-testis-enriched |
| Cytoskeleton remodeling_TGF, WNT and cytoskeletal remodeling | MT-testis-enriched |
| Immune response_MIF-induced cell adhesion, migration and angiogenesis | MT-testis-enriched |
| Protein folding_Membrane trafficking and signal transduction of G-alpha (i) heterotrimeric G-protein | MT-testis-enriched |
| Cell cycle_Start of DNA replication in early S phase | Ovary-enriched |
| Cell cycle_ESR1 regulation of G1/S transition | Ovary-enriched |
| Development_WNT signaling pathway. Part 2 | Ovary-enriched |
| Development_Regulation of telomere length and cellular immortalization | Ovary-enriched |
| Development_WNT signaling pathway. Part 1. Degradation of beta-catenin in the absence WNT signaling | Ovary-enriched |
| Mitogenic action of Estradiol / ESR1 (nuclear) in breast cancer | Ovary-enriched |
| Transcription_CoREST complex-mediated epigenetic gene silencing | Ovary-enriched |
| Cell cycle_Cell cycle (generic schema) | Ovary-enriched |
| Transcription_Ligand-dependent activation of the ESR1/SP pathway | Ovary-enriched |
| G-protein signaling_RhoA regulation pathway | Ovary-enriched |

Table 4 The top ten GeneGo pathways enriched among genes significantly differentially expressed between 40 dpf methytestosterone-treated testes and 40 dpf control testes.

| Pathways |  |
| --- | --- |
| Cell cycle_Initiation of mitosis | MT-testis-enriched |
| Cell cycle_Role of APC in cell cycle regulation | MT-testis-enriched |
| Cell cycle_Spindle assembly and chromosome separation | MT-testis-enriched |
| Cell cycle_Nucleocytoplasmic transport of CDK/Cyclins | MT-testis-enriched |
| Transport_RAN regulation pathway | MT-testis-enriched |
| dGTP metabolism | MT-testis-enriched |
| TTP metabolism | MT-testis-enriched |
| Cell cycle_Chromosome condensation in prometaphase | MT-testis-enriched |
| Cell cycle_Sister chromatid cohesion | MT-testis-enriched |
| dCTP/dUTP metabolism | MT-testis-enriched |
| Transcription_P53 signaling pathway | Testis-enriched |
| DNA damage_Inhibition of telomerase activity and cellular senescence | Testis-enriched |
| Cortisol biosynthesis from Cholesterol | Testis-enriched |
| Transcription_Androgen Receptor nuclear signaling | Testis-enriched |
| Cortisone biosynthesis and metabolism | Testis-enriched |
| Cell adhesion_Endothelial cell contacts by junctional mechanisms | Testis-enriched |
| Cell cycle_Role of SCF complex in cell cycle regulation | Testis-enriched |
| DNA damage_Brca1 as a transcription regulator | Testis-enriched |
| Apoptosis and survival_Granzyme A signaling | Testis-enriched |
| Cell cycle_Role of SCF complex in cell cycle regulation | Testis-enriched |

Table 5 The top ten GeneGo pathways enriched among genes significantly expressed between 60 dpf methytestosterone-treated testes and 60 dpf control ovaries.

| Pathways |  |
| --- | --- |
| Ovarian cancer (main signaling cascades) | MT-testis-enriched |
| Oxidative phosphorylation | MT-testis-enriched |
| Immune response_MIF-JAB1 signaling | MT-testis-enriched |
| Cell cycle_Role of Nek in cell cycle regulation | MT-testis-enriched |
| Stimulation of TGF-beta signaling in lung cancer | MT-testis-enriched |
| Cell cycle_Spindle assembly and chromosome separation | MT-testis-enriched |
| Cell cycle_Nucleocytoplasmic transport of CDK/Cyclins | MT-testis-enriched |
| Cell cycle_Cell cycle (generic schema) | MT-testis-enriched |
| Cytoskeleton remodeling_TGF, WNT and cytoskeletal remodeling | MT-testis-enriched |
| Immune response_IL-1 signaling pathway | MT-testis-enriched |
| Cell cycle_Initiation of mitosis | Ovary-enriched |
| Cytoskeleton remodeling_TGF, WNT and cytoskeletal remodeling | Ovary-enriched |
| Neurophysiological process_Receptor-mediated axon growth repulsion | Ovary-enriched |
| Development_WNT signaling pathway. Part 2 | Ovary-enriched |
| Reproduction_Progesterone-mediated oocyte maturation | Ovary-enriched |
| Cell adhesion_Integrin-mediated cell adhesion and migration | Ovary-enriched |
| DeltaF508-CFTR traffic / ER-to-Golgi in CF | Ovary-enriched |
| wtCFTR traffic / ER-to-Golgi (normal) | Ovary-enriched |
| G-protein signaling_RhoA regulation pathway | Ovary-enriched |
| Cell adhesion_Chemokines and adhesion | Ovary-enriched |

Table 6 The top ten GeneGo pathways enriched among genes significantly expressed between 60 dpf methytestosterone-treated testes and 60 dpf control testes.

| Pathways |  |
| --- | --- |
| Cell cycle_Start of DNA replication in early S phase | Testis-enriched |
| Muscle contraction_Relaxin signaling pathway | Testis-enriched |
| Unsaturated fatty acid biosynthesis | Testis-enriched |
| n-3 Polyunsaturated fatty acid biosynthesis | Testis-enriched |
| n-6 Polyunsaturated fatty acid biosynthesis | Testis-enriched |
| dCTP/dUTP metabolism | Testis-enriched |
| dATP/dITP metabolism | Testis-enriched |
| ATP metabolism | Testis-enriched |
| CTP/UTP metabolism | Testis-enriched |
| ATP/ITP metabolism | Testis-enriched |
