# Supplementary material for: Histological and transcriptomic effects of 17α-methyltestosterone on zebrafish gonad development
Source: BMC Genomics. 2017 Jul 24;18:557. doi: 10.1186/s12864-017-3915-z (PMC5523153; doi:10.1186/s12864-017-3915-z)
Supplement: Supplementary file 10 — Results of qRT-PCR validation of RNA-Seq data. The RNA-seq results are expressed in terms of normalized fold-change (adjusted p-value < 0.05) while qPCR data are expressed as the average relative fold change between samples normalised against the eef1a1l1 reference gene. Fold change values are compared against the 40 dpf control ovary group (40CO) and 60 dpf control ovary group (60CO). (DOCX 17 kb) [file 12864_2017_3915_MOESM10_ESM.docx]

**Table 1. qPCR validation of candidate genes for 40 dpf zebrafish.**

The RNA-seq results are expressed in terms of normalized fold-change (adjusted p-value < 0.05) while qPCR data are expressed as the average relative fold change between samples normalised against the eef1a1l1 reference gene. Fold change values are compared against the 40 dpf control ovary group (40CO).

| 40 dpf |  |  | RNA-seq |  | qPCR |  |
| --- | --- | --- | --- | --- | --- | --- |
| Gene symbol | **Gene ID** | **Description** | **40CT/40CO** | **40MT/40CO** | **40CT/40CO** | **40MT/40CO** |
| *gsdf* | NM_001114668.1 | gonadal somatic cell derived factor | 141.09 | 115.21 | 5.86 | 6.04 |
| *amh* | NM_001007779.1 | anti-Mullerian hormone | 129.92 | 66.56 | 9.25 | 9.4 |
| *star* | NM_131663.1 | steroidogenic acute regulatory protein | 70.58 | 22.17 | 7.73 | 6.83 |
| *cyp11c1* | NM_001080204.1 | cytochrome P450, family 11, subfamily C, polypeptide 1 | 40.77 | 8.8 | 10.67 | 8.81 |
| *hormad1* | NM_001002357.1 | HORMA domain containing 1 | 37.51 | 84.04 | 6.39 | 7.78 |
| *sycp3l* | NM_001040350.1 | synaptonemal complex protein 3 | 22.24 | 57.81 | 4.51 | 6.48 |
| *dmrt1* | NM_205628.1 | doublesex and mab-3 related transcription factor 1 | 13.89 | 10 | 3.67 | 3.71 |
| *wt1a* | NM_131046.1 | wilms tumor 1a | 7.68 | 4.34 | 3.56 | 4.1 |
| *bmp15* | NM_001020484.1 | bone morphogenetic protein 15 | -13.76 | -8.35 | -5.96 | -4.41 |
| *figla* | NM_198919.2 | factor in the germline alpha | -17.47 | -7.76 | -5.12 | -3.9 |
| *gdf9* | NM_001012383.1 | growth differentiation factor 9 | -13.76 | -7.13 | -5.59 | -4.27 |
| *lhx8a* | NM_001003980.2 | LIM homeobox 8a | -7.81 | -5.74 | -4.12 | -3.72 |

**Table 2. qPCR validation of candidate genes for 60 dpf zebrafish.**

The RNA-seq results are expressed in terms of normalized fold-change (adjusted p-value < 0.05) while qPCR data are expressed as the average relative fold change between samples normalised against the eef1a1l1 reference gene. Fold change values are compared against the 60 dpf control ovary group (60CO).

| 60 dpf |  |  | RNA-Seq | | qPCR |  |
| --- | --- | --- | --- | --- | --- | --- |
| Gene symbol | **Gene ID** | **Description** | **60CT/60CO** | **60MT/60CO** | **60CT/60CO** | **60MT/60CO** |
| *gsdf* | NM_001114668.1 | gonadal somatic cell derived factor | 69.01 | 120.57 | 6.25 | 7.18 |
| *amh* | NM_001007779.1 | anti-Mullerian hormone | 72.07 | 74.09 | 6.35 | 7.3 |
| *star* | NM_131663.1 | steroidogenic acute regulatory protein | 117.91 | 83.7 | 8.57 | 4.68 |
| *cyp11c1* | NM_001080204.1 | cytochrome P450, family 11, subfamily C, polypeptide 1 | 61.48 | 43.34 | 11.89 | 9.4 |
| *hormad1* | NM_001002357.1 | HORMA domain containing 1 | 108.96 | 181.51 | 8.42 | 6.23 |
| *sycp3l* | NM_001040350.1 | synaptonemal complex protein 3 | 82.59 | 115.7 | 1.64 | 0.44 |
| *dmrt1* | NM_205628.1 | doublesex and mab-3 related transcription factor 1 | 23.43 | 27.38 | 5.07 | 5.16 |
| *wt1a* | NM_131046.1 | wilms tumor 1a | 3.32 | 3.73 | 2.76 | -0.2 |
| *bmp15* | NM_001020484.1 | bone morphogenetic protein 15 | -19.85 | -26.1 | -6.03 | -6.4 |
| *figla* | NM_198919.2 | factor in the germline alpha | -18.88 | -20.07 | -5.84 | -6.89 |
| *gdf9* | NM_001012383.1 | growth differentiation factor 9 | -14.52 | -18.24 | -5.44 | -6.16 |
| *lhx8a* | NM_001003980.2 | LIM homeobox 8a | -13.17 | -18.32 | -5.51 | -6.23 |
